# Prenatal biochemical screening and long term risk of maternal cardiovascular disease: population based cohort study

**DOI:** 10.1136/bmj.k2739

**Published:** 2018-07-11

**Authors:** Joel G Ray, Tianhua Huang, Wendy S Meschino, Eyal Cohen, Alison L Park

**Affiliations:** 1 Departments of Medicine, Health Policy Management and Evaluation, and Obstetrics and Gynecology, St Michael’s Hospital, Toronto, ON, Canada, M5B 1W8; 2 Institute for Clinical Evaluative Sciences, University of Toronto, Toronto, ON, Canada; 3 Genetics Program, North York General Hospital, Toronto, ON, Canada; 4 Institute of Health Policy, Management and Evaluation, University of Toronto, Toronto, ON, Canada; 5 Department of Paediatrics, University of Toronto, Toronto, ON, Canada; 6 Department of Pediatrics, Hospital for Sick Children, Toronto, ON, Canada

## Abstract

**Objective:**

To examine whether abnormal prenatal biochemical screening results are associated with an increased risk of premature cardiovascular disease after pregnancy.

**Design:**

Population based cohort study.

**Setting:**

The entire province of Ontario, Canada, where healthcare is universally available.

**Participants:**

Women aged 12-55 years, without pre-existing cardiovascular disease, who underwent prenatal screening between 1993 and 2011. One pregnancy per woman was randomly selected.

**Exposures:**

Low (≤5th centile multiple of the median) serum total chorionic gonadotropin, unconjugated estriol, and pregnancy associated plasma protein A and high (≥95th centile multiple of the median) alphafetoprotein and dimeric inhibin-A.

**Main outcome measures:**

Composite of hospital admission or revascularisation for coronary artery, cerebrovascular, or peripheral arterial disease or hospital admission for heart failure or dysrhythmia at least 365 days after pregnancy.

**Results:**

Among 855 536 pregnancies, and after a median of 11.4 (interquartile range 6.8-17.5) years of follow-up, 6209 women developed the main cardiovascular disease outcome. Abnormal results for each of the five prenatal biochemical screening analytes, especially dimeric inhibin-A, were associated with a higher risk of cardiovascular disease. Women with an abnormally high dimeric inhibin-A (≥95th centile) had the highest rate of cardiovascular disease (30 events or 8.3 per 10 000 person years versus 251 events or 3.8 per 10 000 person years for those <95th centile; multivariable adjusted hazard ratio 2.0, 95% confidence interval 1.4 to 3.0). Compared with women without any abnormal biochemical measure, the hazard ratio for the cardiovascular disease composite outcome was 1.2-1.3 times higher with one abnormal analyte and 1.5-2.0 times higher with two or more abnormal analytes.

**Conclusions:**

Women with abnormal prenatal biochemical screening results, especially for dimeric inhibin-A, may be at higher risk of cardiovascular disease. If these findings are replicated elsewhere, a massive amount of data exists that could aid in identifying women at higher risk of premature cardiovascular disease and that could be conveyed to them or their healthcare providers.

## Introduction

A healthy pregnancy depends on the successful linkage of a woman’s physiology with that of her fetus, via the placenta. Placental hormones arise from the syncytiotrophoblast and, in combination with cytokines and growth factors, alter various maternal physiological systems as a means to sustaining pregnancy.^1^ ^2^ Placental vascular disease probably arises within the temporary interface of the trophoblast and endometrial decidua, resulting in adversity for mother and fetus, including pre-eclampsia, placental abruption, poor fetal growth, and preterm birth.^3^

Several risk factors for placental vascular disease, especially for pre-eclampsia,^3^ ^4^ are the same as those for cardiovascular disease (see supplementary file 1). A maternal placental syndrome in pregnancy seems to forecast a woman’s cardiovascular health in the years that follow, including premature onset before age 65 years of coronary artery disease,^5^ ^6^ heart failure, and dysrhythmias,^7^ as well as death after coronary revascularisation.^8^ Different guidelines for the prevention of cardiovascular disease recommend screening for risk factors in women with a previous maternal placental syndrome.^9^

Starting around 1993, maternal serum screening was made freely available to all pregnant women in Ontario, to screen for trisomies 21 and 18 and neural tube defects. Triple screening comprised maternal serum alphafetoprotein, total human chorionic gonadotropin, and unconjugated estriol, collected in the second trimester at 15^+0^ to 20^+6^ weeks’ gestation. Thereafter, dimeric inhibin-A was added. By 2000, serum pregnancy associated plasma protein A, measured at 11^+0^ to 13^+6^ weeks’, had also been added. Although maternal serum screening has been used primarily to detect anomalies in the fetus, a particular pattern of prenatal biochemical screening results—a high serum alphafetoprotein, human chorionic gonadotropin, or dimeric inhibin-A and low unconjugated estriol or pregnancy associated plasma protein A—has been found to have a high specificity for identifying women at risk of pre-eclampsia in the index pregnancy.^10^

Given that certain prenatal biochemical screening hormones are associated with pre-eclampsia, and that pre-eclampsia is associated with a higher future risk of cardiovascular disease in women, one ensuing question is whether abnormal concentrations of prenatal hormones are associated with a higher risk of cardiovascular disease after pregnancy. The goal of this study was to answer that question. The conceptual framework of the study is summarised in supplementary file 1.

## Methods

This population based cohort study was conducted in Ontario, Canada, where universal healthcare includes prenatal screening and obstetrics care. All prenatal biochemical screening records were eligible and aggregated within the Ontario Maternal Multiple Marker Screening Database for 1993 to 2011. The uptake of prenatal screening varies geographically, between 28% and 80%.^11^ We linked screened pregnancies in the Ontario Maternal Multiple Marker Screening Database to administrative health databases at the Institute for Clinical Evaluation Sciences (ICES), using each woman’s unique encoded identifiers. Specifics about the ICES databases are described elsewhere and shown in supplementary file 2.^6^ ^7^ ^8^

### Participants

We included female patients aged 12-55 years who underwent prenatal biochemical screening at 11 to 20 weeks’ gestation during 1993-2011. We included them regardless of the outcome of pregnancy (that is, miscarriage or ectopic pregnancy at 11 to <20 weeks, induced abortion at 11 to <20 weeks, stillbirth ≥20 weeks, or live birth ≥20 weeks of gestation). We excluded women diagnosed as having any cardiac, cerebrovascular, or peripheral arterial disease five years or less before the prenatal biochemical screening in the index pregnancy (supplementary file 2). We also excluded non-Ontarian residents and those without a valid OHIP health card number (supplementary file 2). Of all remaining deliveries, we randomly selected one pregnancy per woman as the index pregnancy to simplify the data analyses. These pregnancies formed the screened cohort. Recognised pregnancies without prenatal biochemical screening were assembled in a non-screened cohort and analysed in a supplementary manner, as outlined below.

### Exposures and outcomes

The exposure of interest was each prenatal biochemical screening analyte— alphafetoprotein, human chorionic gonadotropin, unconjugated estriol, dimeric inhibin-A, and pregnancy associated plasma protein A. As the last two were added to prenatal screening in later years, the number of different analytes for each pregnancy could vary. The unit of analysis for each analyte was its multiple of the median, a convention commonly used in clinical reporting that standardises test results between different laboratories, describing how far an individual test result deviates from the median concentration at a given gestational age. Derivation of the multiple of the median cut-off points used to define “abnormal” for each prenatal biochemical screening analyte is described below.

The primary outcome was a cardiovascular disease composite of any hospital admission or revascularisation for coronary artery disease, cerebrovascular disease, or peripheral arterial disease or any hospital admission for heart failure or dysrhythmia, arising at least 365 days after the start of the index pregnancy (“time zero”) (supplementary file 2). To establish a common starting point for the follow-up period for each participant, we calculated “time zero” by subtracting the gestational age (in days) at prenatal screening from the date at prenatal screening—equivalent to the estimated first day of the last menstrual period—and then added 365 days to that date. Starting follow-up at 365 days ensured that a woman was well past her index pregnancy and avoided including within the cardiovascular disease composite an event that was a direct consequence of a complication of pregnancy, such as peripartum stroke or heart failure due to pre-eclampsia. Censoring was on death or arrival at the end of the study (31 March 2016).

The secondary outcome was major adverse cardiovascular events, comprising all cause mortality or any hospital admission for myocardial infarction or stroke, arising at least 365 days after the start of the index pregnancy, without censoring on death.

We used the international classification of diseases (ICD) coding system (ICD-9 before 2002 and ICD-10-CA thereafter) to identify all study outcomes. Several outcomes have been validated under this approach (supplementary file 2, last column). We identified maternal mortality from the Ontario Ministry of Health and Long Term Care’s Registered Persons Database. Fifths of dissemination area income and rurality were based on Statistics Canada census data.

### Statistical analysis

We explored the shape of the association between each continuous analyte (in multiple of the median) and the log hazard of cardiovascular disease by using univariable fractional polynomial regression and the RA2 selection algorithm, which selected the best fitting of 44 regression models with different combinations of power transformations of the explanatory variable.^12^ ^13^ Inspection of the derived plots showed extreme outliers of multiple of the median well beyond the 0.2nd or 99.8th centiles, probably related to pregnancies affected by an anomaly,^14^ which was not resolved by various mathematical transformations of the multiple of the median.^15^ ^16^ After removal of live births or stillbirths with a diagnosis of a congenital or chromosomal anomaly on the maternal or newborn hospital record, as well as outliers of multiple of the median beyond the 0.2nd or 99.8th centiles from the fractional polynomial models (but not from the study cohort),^17^ the best fit plots were more interpretable (supplementary file 3). Inspection of each plot, while considering the existing literature related to placental disease, such as pre-eclampsia,^10^ ^18^ facilitated the setting of the abnormal cut-off points at the 5th centile multiple of the median for human chorionic gonadotropin, unconjugated estriol, and pregnancy associated plasma protein A and at the 95th centile multiple of the median for alphafetoprotein and dimeric inhibin-A. The comparative referent for each analyte was a multiple of the median above the 5th centile for the first three analytes and below the 95th centile for the last two.

The main model assessed the primary cardiovascular disease composite outcome, as well as the individual outcomes of coronary artery disease and cerebrovascular disease, in relation to each analyte for the screened cohort, with censoring at a woman’s death or arrival at the end of the study period of 31 March 2016, allowing for a maximum follow-up of 22 years. We did time to event analyses using multivariable Cox regression models, to derive a hazard ratio and 95% confidence interval for each study outcome. Assessment of the secondary outcome of major adverse cardiovascular events was not censored on death. Hazard ratios were adjusted for variables chosen a priori, based on the existing literature,^3^ ^4^ ^5^ ^6^ ^7^ including maternal age (continuous), gravidity (1, ≥2, missing), fifth of neighbourhood income (1, 2, 3, 4, 5, missing), rural residence (rural, urban, missing), ethnicity (Asian, oriental, black, white, Hispanic, other, missing), and gestational age (continuous), each at the time of prenatal biochemical screening, as well as maternal diabetes mellitus, chronic hypertension, renal disease, tobacco/illicit drug use, and dyslipidaemia within 365 days before or after the start of the index pregnancy (that is, within two years before time zero). “Asian” and “oriental” were classified together in some years, as were “Hispanic” and “other.” As maternal weight at the time of screening was missing for about 10% of pregnancies, we adjusted for it in an analysis restricted to women with non-missing weight (additional analysis 1). We assessed the proportional hazards assumption by using a Wald test for interaction between the exposure and a function of survival time, which did not detect a significant departure.

We further assessed the cardiovascular disease composite outcome in relation to the number of abnormal prenatal biochemical screening analytes: none (referent), one, or two or more (additional analysis 2). As pregnancy associated plasma protein A and dimeric inhibin-A were unavailable in the earlier years, we did this analysis separately for specimens from 1993 to 2002 and those from 2003 to 2011. To increase the specificity of the exposure, we also ran the main model for the cardiovascular disease composite outcome by using abnormal cut-off points of the 1st centile multiple of the median for human chorionic gonadotropin, unconjugated estriol, and pregnancy associated plasma protein A and the 99th centile multiple of the median for alphafetoprotein and dimeric inhibin-A (additional analysis 3).

In additional analysis 4, we re-evaluated each biochemical analyte in the absence or co-presence of each of five factors known to be associated with maternal cardiovascular disease: any chromosomal or congenital anomaly at the time of a live birth or stillbirth—a reflection of a higher likelihood of having abnormal biochemical screening, chronic maternal stress, and unmeasurable genetic factors in both mother and child; preterm birth before 37 weeks’ gestation at the time of a live birth^2^; a pregnancy ending in a non-live birth^6^; a maternal placental syndrome—pre-eclampsia, gestational hypertension, or placental abruption or infarction^6^ ^7^—at the time of a live birth or a stillbirth; and advanced maternal age (≥35 years). In each model, the referent was a normal biochemical measure in the absence of the given perinatal or maternal factor.

We re-ran the main model for the cardiovascular disease composite outcome, restricting it to a sub-set of women with two or more pregnancies with prenatal screening. The hazard ratios were also adjusted for the number of previous pregnancies with a given abnormal serum analyte (additional analysis 5).

A woman who undergoes prenatal screening may differ from one who does not. To account for this, from among all women in the non-screened cohort (and who also met the same criteria as for the screened cohort (supplementary file 2)), we randomly selected non-screened women and matched them 1:1 by year of pregnancy to counterparts in the screened cohort. We compared baseline variables between screened and non-screened cohorts by using standardised differences and expressed the primary cardiovascular disease composite outcome as an adjusted hazard ratio, accounting for the matching in the aforementioned Cox regression model (additional analysis 6).

We used SAS version 9.4 for UNIX for all statistical analyses.

### Patient involvement

No patients were involved in setting the research question or the outcome measures, nor were they involved in developing plans for the design or implementation of the study. No patients were asked to advise on interpretation or writing up of results. There are no plans to disseminate the results of the research to study participants or the relevant patient community.

## Results

We identified 1 380 840 pregnancies in the Ontario Maternal Multiple Marker Screening Database during the study period, of which 1 210 146 (87.6%) formed the screened cohort (supplementary file 4). For each given analyte, we randomly selected one pregnancy per woman to analyse, resulting in 855 536 included pregnancies (supplementary file 4). Serum alphafetoprotein was the most frequent analyte (807 292 pregnancies), whereas dimeric inhibin-A was available only after 2002 (91 826 pregnancies) (table 1). The mean maternal age was about 30 years, around 35% of participants were non-white, and 43% were primigravid. Nearly 97% of recognised pregnancies ended in a live birth (table 1). The rate of diabetes mellitus was about 5.5%, chronic hypertension 3.4%, dyslipidaemia 1.1%, renal disease 0.3%, and drug dependence/tobacco use 1.4%. The selected 95th and 99th centile multiple of the median cut-off points for each analyte are listed at the bottom of table 1. The overall median duration of follow-up was 11.4 (interquartile range 6.8-17.5) years, varying from 12.9 (7.9-18.1) years in the human chorionic gonadotropin group to 7.1 (5.0-9.5) years in the pregnancy associated plasma protein A group (table 1). We had 10 358 377 person years of follow-up among all randomly selected pregnancies, largely among women with measured alphafetoprotein, human chorionic gonadotropin, and unconjugated estriol (table 1).

**Table 1:** Characteristics of pregnancies with prenatal biochemical screening, by analyte. One randomly selected pregnancy per woman was formed study cohort. Values are numbers (percentages) unless stated otherwise

Characteristics	Biochemical serum screening analyte (No of exclusive pregnancies)					
	Any analyte (n=855 536)	Alphafetoprotein (n=807 292)	Unconjugated estriol (n=799 363)	Total human chorionic gonadotropin (n=784 627)	Pregnancy associated plasma protein A (n=393 399)	Dimeric inhibin-A (n=91 826)
**Characteristics at time of maternal serum screening**						
Mean (SD) age, years	29.9 (5.3)	29.9 (5.3)	29.8 (5.2)	29.8 (5.2)	30.8 (5.2)	29.1 (5.6)
Advanced maternal age, 35-39 years	143 682 (16.8)	132 680 (16.4)	126 892 (15.9)	124 617 (15.9)	82 374 (20.9)	13 442 (14.6)
Advanced maternal age, 40-44 years	22 807 (2.7)	19 720 (2.4)	18 245 (2.3)	17 812 (2.3)	14 806 (3.8)	2422 (2.6)
Advanced maternal age, 45-55 years	806 (0.1)	670 (0.1)	581 (0.1)	560 (0.1)	549 (0.1)	104 (0.1)
Ethnicity*:						
White	549 070 (64.2)	514 882 (63.8)	515 155 (64.4)	502 408 (64.0)	245 922 (62.5)	46 502 (50.6)
Asian	188 800 (22.1)	180 745 (22.4)	177 593 (22.2)	176 409 (22.5)	96 274 (24.5)	27 268 (29.7)
Black	49 640 (5.8)	47 217 (5.8)	46 376 (5.8)	46 093 (5.9)	20 447 (5.2)	8141 (8.9)
Other	18 012 (2.1)	16 204 (2.0)	16 093 (2.0)	15 602 (2.0)	12 181 (3.1)	4122 (4.5)
Oriental	48 370 (5.7)	1647 (0.2)	1604 (0.2)	1616 (0.2)	0.0 (0.0)	0.0 (0.0)
Unknown	549 070 (64.2)	46 597 (5.8)	42 542 (5.3)	42 499 (5.4)	18 575 (4.7)	5793 (6.3)
Fifth of income:						
1 (lowest)	194 255 (22.7)	183 584 (22.7)	182 157 (22.8)	179 164 (22.8)	77 155 (19.6)	27 097 (29.5)
5 (highest)	140 074 (16.4)	130 733 (16.2)	128 515 (16.1)	125 897 (16.0)	72 229 (18.4)	10 306 (11.2)
Missing	3228 (0.4)	3018 (0.4)	2955 (0.4)	2907 (0.4)	1108 (0.3)	566 (0.6)
Rural residence	61 811 (7.2)	58 090 (7.2)	59 562 (7.5)	57 191 (7.3)	21 229 (5.4)	8683 (9.5)
Median (interquartile range) gravidity	2 (1-3)	2 (1-3)	2 (1-3)	2 (1-3)	2 (1-2)	2 (1-3)
Primigravid	365 582 (42.7)	344 681 (42.7)	340 154 (42.6)	334 252 (42.6)	166 716 (42.4)	38 093 (41.5)
Gravidity unknown	13 044 (1.5)	10 164 (1.3)	10 033 (1.3)	9675 (1.2)	10 125 (2.6)	1861 (2.0)
Mean (SD) maternal weight, kg	64.4 (18.0)	65.3 (17.1)	65.4 (17.1)	65.3 (17.1)	60.0 (20.9)	63.9 (19.7)
Missing maternal weight	85 423 (10.0)	79 549 (9.9)	74 956 (9.4)	74 807 (9.5)	65 946 (16.8)	12 363 (13.5)
Type of pregnancy:						
Singleton	787 147 (92.0)	767 743 (95.1)	768 160 (96.1)	753 334 (96.0)	339 403 (86.3)	90 732 (98.8)
Multi-fetal	9624 (1.1)	9726 (1.2)	5059 (0.6)	5036 (0.6)	144 (0.0)	149 (0.2)
Unknown	58 765 (6.9)	29 823 (3.7)	26 144 (3.3)	26 257 (3.3)	53 852 (13.7)	945 (1.0)
Mean (SD) gestational age at screening, weeks	16.4 (1.5)	16.7 (1.1)	16.7 (1.1)	16.7 (1.1)	12.5 (0.5)	17.0 (1.3)
Year of screening:						
1993-2002	389 406 (45.5)	391 549 (48.5)	386 415 (48.3)	386 952 (49.3)	14 926 (3.8)	0
2003-11	466 130 (54.5)	415 743 (51.5)	412 948 (51.7)	397 675 (50.7)	378 473 (96.2)	91 826 (100.0)
**Other characteristics**						
Outcome of index pregnancy:						
Live birth	823 787 (96.3)	780 657 (96.7)	773 164 (96.7)	758 779 (96.7)	376 849 (95.8)	88 681 (96.6)
Stillbirth	4161 (0.5)	4040 (0.5)	3820 (0.5)	3844 (0.5)	1664 (0.4)	557 (0.6)
Miscarriage	3616 (0.4)	2412 (0.3)	2358 (0.3)	2348 (0.3)	2260 (0.6)	338 (0.4)
Induced abortion	3245 (0.4)	2200 (0.3)	2194 (0.3)	2180 (0.3)	2501 (0.6)	389 (0.4)
Unknown outcome	20 727 (2.4)	17 983 (2.2)	17 827 (2.2)	17 476 (2.2)	10 125 (2.6)	1861 (2.0)
Conditions ≤365 days before, or up to 365 days after, start of pregnancy:						
Diabetes mellitus	46 78 (5.5)	44 539 (5.5)	43 832 (5.5)	43 011 (5.5)	22 231 (5.7)	5195 (5.7)
Chronic hypertension	29 327 (3.4)	27 154 (3.4)	26 734 (3.3)	26 149 (3.3)	15 933 (4.1)	3534 (3.8)
Dyslipidaemia	9777 (1.1)	9296 (1.2)	9008 (1.1)	8856 (1.1)	5753 (1.5)	1424 (1.6)
Renal disease	2301 (0.3)	2106 (0.3)	2068 (0.3)	2021 (0.3)	1282 (0.3)	323 (0.4)
Drug dependence/tobacco use	11 589 (1.4)	10 597 (1.3)	10 605 (1.3)	10 246 (1.3)	5350 (1.4)	1531 (1.7)
Conditions at live birth or stillbirth delivery:	(n=827 948)	(n=784 697)	(n=776 84)	(n=762 623)	(n=378 513)	(n=89 238)
Congenital or chromosomal anomaly	31 674 (3.8)	30 470 (3.9)	30 182 (3.9)	29 589 (3.9)	11 665 (3.1)	2479 (2.8)
Pre-eclampsia or eclampsia	16 604 (2.0)	16 023 (2.0)	15 691 (2.0)	15 412 (2.0)	4295 (1.1)	968 (1.1)
Gestational hypertension	27 196 (3.3)	25 254 (3.2)	24 884 (3.2)	24 258 (3.2)	15 763 (4.2)	3406 (3.8)
Placental abruption	7601 (0.9)	7237 (0.9)	7126 (0.9)	7079 (0.9)	2940 (0.8)	771 (0.9)
Placental infarction	4959 (0.6)	4878 (0.6)	4740 (0.6)	4732 (0.6)	1451 (0.4)	268 (0.3)
Preterm live birth <37 weeks’ gestation	57 481 (6.9)	54 291 (6.9)	51 219 (6.6)	50 306 (6.6)	25 109 (6.6)	6014 (6.7)
Prenatal biochemical serum screening analyte†:						
No of pregnancies	–	1 055 118	1 045 859	1 024 401	473 091	93 518
95th centile MoM cut-off point	–	1.83	0.49	0.51	0.38	2.20
99th centile MoM cut-off point	–	2.52	0.32	0.34	0.25	3.21
Median (interquartile range) years of follow-up, from ≥365 days after start of index pregnancy	11.4 (6.8-17.5)	12.3 (7.0-17.9)	11.8 (6.8-17.6)	12.9 (7.9-18.1)	7.1 (5.0-9.5)	8.5 (7.3-9.7)
Total person years of follow-up, from ≥365 days after start of index pregnancy	10 358 377	10 074 600	9 936 960	9 878 639	2 928 17	692 75

MoM=multiple of the median.

* In some years, “Asian” and “oriental” were classified together.

† Cut-off points were derived from pregnancies resulting in live birth or stillbirth without diagnosis of congenital or chromosomal anomaly at time of index birth.

A total of 6209 women developed the primary cardiovascular disease composite outcome, which was typically about 1.2 to 1.3 times more likely to occur in a pregnancy with an abnormal biochemical analyte, even after adjustment for other covariates (table 2). However, women with an abnormally elevated dimeric inhibin-A had a more pronounced rate of the cardiovascular disease composite outcome (8.3 per 10 000 person years) than those below the cut-off point (3.8 per 10 000 person years), equivalent to an adjusted hazard ratio of 2.0 (95% confidence interval 1.4 to 3.0) (upper part of table 2). These findings were not altered by adjustment for maternal weight (additional analysis 1, supplementary file 5), nor by the exclusion of women with pre-existing renal disease, chronic hypertension, or dyslipidaemia (supplementary file 6).

**Table 2:** Risk of primary cardiovascular disease composite outcome of any hospital admission or revascularisation for coronary artery, cerebrovascular, or peripheral arterial disease or any hospital admission for heart failure or dysrhythmia, arising ≥365 days after start of index pregnancy (upper part), coronary artery disease outcome arising ≥365 days after start of index pregnancy (middle part), and cerebrovascular disease outcome arising ≥365 days after start of index pregnancy (lower part), each in association with an abnormal cut-off point of 5th or 95th centile of multiple of the median (MoM) for given serum analyte

Abnormal serum analyte and cut-off points used to define normal and abnormal	No (incidence rate) per 10 000 person years	Unadjusted hazard ratio (95% CI)	Adjusted hazard ratio (95% CI)*
**Cardiovascular disease composite outcome**			
High alphafetoprotein:			
Normal: ≤95th centile MoM (n=763 716)	5600 (5.9)	1.0 (reference)	1.0 (reference)
Abnormal: >95th centile MoM (n=43 576)	420 (7.4)	1.2 (1.1 to 1.3)	1.2 (1.1 to 1.3)
Low total human chorionic gonadotropin:			
Normal: ≥5th centile MoM (n=741 491)	5670 (6.0)	1.0 (reference)	1.0 (reference)
Abnormal: <5th centile MoM (n=43 136)	224 (5.8)	1.3 (1.2 to 1.4)	1.2 (1.1 to 1.4)
Low unconjugated estriol:			
Normal: ≥5th centile MoM (n=756 958)	5332 (5.8)	1.0 (reference)	1.0 (reference)
Abnormal: <5th centile MoM (n=42 405)	585 (8.5)	1.3 (1.1 to 1.5)	1.3 (1.2 to 1.4)
High dimeric inhibin-A:			
Normal: ≤95th centile MoM (n=87 097)	251 (3.8)	1.0 (reference)	1.0 (reference)
Abnormal: >95th centile MoM (n=4729)	30 (8.3)	2.2 (1.5 to 3.2)	2.0 (1.4 to 3.0)
Low pregnancy associated plasma protein A:			
Normal: ≥5th centile MoM (n=371 097)	990 (3.6)	1.0 (reference)	1.0 (reference)
Abnormal: <5th centile MoM (n=22 302)	84 (5.1)	1.4 (1.1 to 1.8)	1.3 (1.1 to 1.7)
**Coronary artery disease outcome**			
High alphafetoprotein:			
Normal: ≤95th centile MoM (n=763 716)	3024 (3.2)	1.0 (reference)	1.0 (reference)
Abnormal: >95th centile MoM (n=43 576)	237 (4.2)	1.3 (1.1 to 1.4)	1.2 (1.1 to 1.4)
Low total human chorionic gonadotropin:			
Normal: ≥5th centile MoM (n=741 491)	3088 (3.2)	1.0 (reference)	1.0 (reference)
Abnormal: <5th centile MoM (n=43 136)	112 (2.9)	1.3 (1.0 to 1.5)	1.2 (1.0 to 1.4)
Low unconjugated estriol:			
Normal: ≥5th centile MoM (n=756 958)	2845 (3.1)	1.0 (reference)	1.0 (reference)
Abnormal: <5th centile MoM (n=42 405)	356 (5.2)	1.4 (1.3 to 1.6)	1.4 (1.3 to 1.6)
High dimeric inhibin-A:			
Normal: ≤95th centile MoM (n=87 097)	127 (1.9)	1.0 (reference)	1.0 (reference)
Abnormal: >95th centile MoM (n=4729)	10 (2.8)	1.4 (0.8 to 2.7)	1.3 (0.7 to 2.4)
Low pregnancy associated plasma protein A:			
Normal: ≥5th centile MoM (n=371 097)	455 (1.6)	1.0 (reference)	1.0 (reference)
Abnormal: <5th centile MoM (n=22 302)	47 (2.8)	1.7 (1.3 to 2.3)	1.6 (1.2 to 2.1)
**Cerebrovascular disease outcome**			
High alphafetoprotein:			
Normal: ≤95th centile MoM (n=763 716)	1219 (1.3)	1.0 (reference)	1.0 (reference)
Abnormal: >95th centile MoM (n=43 576)	100 (1.8)	1.3 (1.1 to 1.6)	1.3 (1.0 to 1.6)
Low total human chorionic gonadotropin:			
Normal: ≥5th centile MoM (n=741 491)	1224 (1.3)	1.0 (reference)	1.0 (reference)
Abnormal: <5th centile MoM (n=43 136)	57 (1.5)	1.5 (1.1 to 1.9)	1.4 (1.1 to 1.8)
Low unconjugated estriol:			
Normal: ≥5th centile MoM (n=756 958)	1181 (1.3)	1.0 (reference)	1.0 (reference)
Abnormal: <5th centile MoM (n=42 405)	109 (1.6)	1.1 (0.9 to 1.3)	1.1 (0.9 to 1.4)
High dimeric inhibin-A:			
Normal: ≤95th centile MoM (n=87 097)	50 (0.8)	1.0 (reference)	1.0 (reference)
Abnormal: >95th centile MoM (n=4729)	12 (3.3)	4.3 (2.3 to 8.1)	3.8 (2.0 to 7.2)
Low pregnancy associated plasma protein A:			
Normal: ≥5th centile MoM (n=371 097)	232 (0.8)	1.0 (reference)	1.0 (reference)
Abnormal: <5th centile MoM (n=22 302)	17 (1)	1.2 (0.7 to 2)	1.2 (0.7 to 1.9)

* Adjusted for maternal age (continuous), gravidity (1, ≥2, missing), fifth of neighbourhood income (1, 2, 3, 4, 5, missing), rural residence (rural, urban, missing), ethnicity (Asian, black, white, Hispanic, oriental, other, missing), and gestational age (continuous), each at time of prenatal biochemical screening, as well as maternal diabetes mellitus, chronic hypertension, renal disease, tobacco/illicit drug use, and dyslipidaemia within 365 days preceding start of index pregnancy, up to and including 365 days after start of index pregnancy (time zero); censored on death or arrival at end of study date of 31 March 2016.

Most cardiovascular disease events were coronary artery (3334, 53.7%) or cerebrovascular (1361, 21.9%) in nature (supplementary file 7). In pregnancies with an abnormal biochemical analyte, a similar pattern to that for any cardiovascular disease was also apparent for coronary artery disease (middle part of table 2) and cerebrovascular disease (lower part of table 2), with an even more pronounced risk for the latter in relation to an abnormal dimeric inhibin-A (adjusted hazard ratio 3.8, 2.0 to 7.2). Women with an abnormal serum analyte were also more likely to have the secondary outcome of major adverse cardiovascular events (table 3). Women with a high dimeric inhibin-A had an adjusted hazard ratio of 1.9 (1.3 to 2.6), and those with a low pregnancy associated plasma protein A had an adjusted hazard ratio of 1.6 (1.3 to 1.9) (table 3).

**Table 3:** Risk of secondary composite outcome of major adverse cardiovascular events, comprising all cause mortality or any hospital admission for myocardial infarction or stroke, arising ≥365 days after start of index pregnancy, in association with abnormal cut-off point of 5th or 95th centile of multiple of the median (MoM) for given serum analyte

Abnormal serum analyte and cut-off points used to define normal and abnormal	Incidence rate per 10 000 person years	Unadjusted hazard ratio (95% CI)	Adjusted hazard ratio (95% CI)*
High alphafetoprotein:			
Normal: ≤95th centile MoM (n=763 716)	7183 (7.5)	1.0 (reference)	1.0 (reference)
Abnormal: >95th centile MoM (n=43 576)	589 (10.3)	1.3 (1.2 to 1.4)	1.3 (1.2 to 1.4)
Low total human chorionic gonadotropin:			
Normal: ≥5th centile MoM (n=741 491)	7291 (7.7)	1.0 (reference)	1.0 (reference)
Abnormal: <5th centile MoM (n=43 136)	291 (7.5)	1.3 (1.1 to 1.4)	1.2 (1.0 to 1.3)
Low unconjugated estriol:			
Normal: ≥5th centile MoM (n=756 958)	6859 (7.4)	1.0 (reference)	1.0 (reference)
Abnormal: <5th centile MoM (n=42 405)	756 (11.0)	1.3 (1.2 to 1.4)	1.3 (1.2 to 1.4)
High dimeric inhibin-A:			
Normal: ≤95th centile MoM (n=87 097)	306 (4.7)	1.0 (reference)	1.0 (reference)
Abnormal: >95th centile MoM (n=4729)	37 (10.3)	2.2 (1.6 to 3.1)	1.9 (1.3 to 2.6)
Low pregnancy associated plasma protein A:			
Normal: ≥5th centile MoM (n=371 097)	1179 (4.3)	1.0 (reference)	1.0 (reference)
Abnormal: <5th centile MoM (n=22 302)	117 (7.0)	1.6 (1.4 to 2.0)	1.6 (1.3 to 1.9)

* Adjusted for maternal age (continuous), gravidity (1, ≥2, missing), fifth of neighbourhood income (1, 2, 3, 4, 5, missing), rural residence (rural, urban, missing), ethnicity (Asian, black, white, Hispanic, oriental, other, missing), and gestational age (continuous), each at time of prenatal biochemical screening, as well as maternal diabetes mellitus, chronic hypertension, renal disease, tobacco/illicit drug use, and dyslipidaemia within 365 days preceding start of index pregnancy, up to and including 365 days after start of index pregnancy (time zero); censored on death or arrival at end of study date of 31 March 2016.

For prenatal screening performed in the earlier era, the risk of the primary cardiovascular disease composite outcome rose with the number of abnormal analytes (additional analysis 2; table 4). For prenatal screening in the later era, when dimeric inhibin-A and pregnancy associated plasma protein A were included, the rate of the primary cardiovascular disease composite outcome was 7.7 per 10 000 person years with two or more abnormal analytes versus 3.6 per 10 000 person years with none, yielding an adjusted hazard ratio of 2.0 (1.4 to 2.8) (additional analysis 2; table 4). Re-setting the respective abnormal cut-off points to the 1st or 99th centile in the main model magnified the hazard ratios for dimeric inhibin-A and pregnancy associated plasma protein A somewhat (additional analysis 3; supplementary file 8).

**Table 4:** Risk of cardiovascular disease composite outcome of any hospital admission or revascularisation for coronary artery, cerebrovascular, or peripheral arterial disease or any hospital admission for heart failure or dysrhythmia, arising ≥365 days after start of index pregnancy, in association with number of abnormal serum analytes, based on 5th or 95th centile of multiple of the median (MoM) cut-off points

No of abnormal serum analytes	Incidence rate per 10 000 person years	Unadjusted hazard ratio (95% CI)	Adjusted hazard ratio (95% CI)*
**1993 to 2002**			
0 (n=326 996)	3828 (6.7)	1.0 (reference)	1.0 (reference)
1 (n=60 203)	926 (8.8)	1.3 (1.2 to 1.4)	1.3 (1.2 to 1.4)
≥2 (n=2207)	42 (10.9)	1.6 (1.2 to 2.2)	1.5 (1.1 to 2.0)
**2003 to 2011**			
0 (n=388 371)	1069 (3.6)	1.0 (reference)	1.0 (reference)
1 (n=72 461)	242 (4.4)	1.2 (1.1 to 1.4)	1.2 (1.0 to 1.4)
≥2 (n=5298)	30 (7.7)	2.2 (1.5 to 3.1)	2.0 (1.4 to 2.8)

As pregnancy associated plasma protein A and dimeric inhibin-A were available only in later years, results are presented separately by era of 1993-2002

and 2003-11.

* Adjusted for maternal age (continuous), gravidity (1, ≥2, missing), fifth of neighbourhood income (1, 2, 3, 4, 5, missing), rural residence (rural, urban, missing), ethnicity (Asian, black, white, Hispanic, oriental, other, missing), and gestational age (continuous), each at time of prenatal biochemical screening, as well as maternal diabetes mellitus, chronic hypertension, renal disease, tobacco/illicit drug use, and dyslipidaemia within 365 days preceding start of index pregnancy, up to and including 365 days after start of index pregnancy (time zero); censored on death or arrival at end of study date of 31 March 2016.

**Fig 1:**
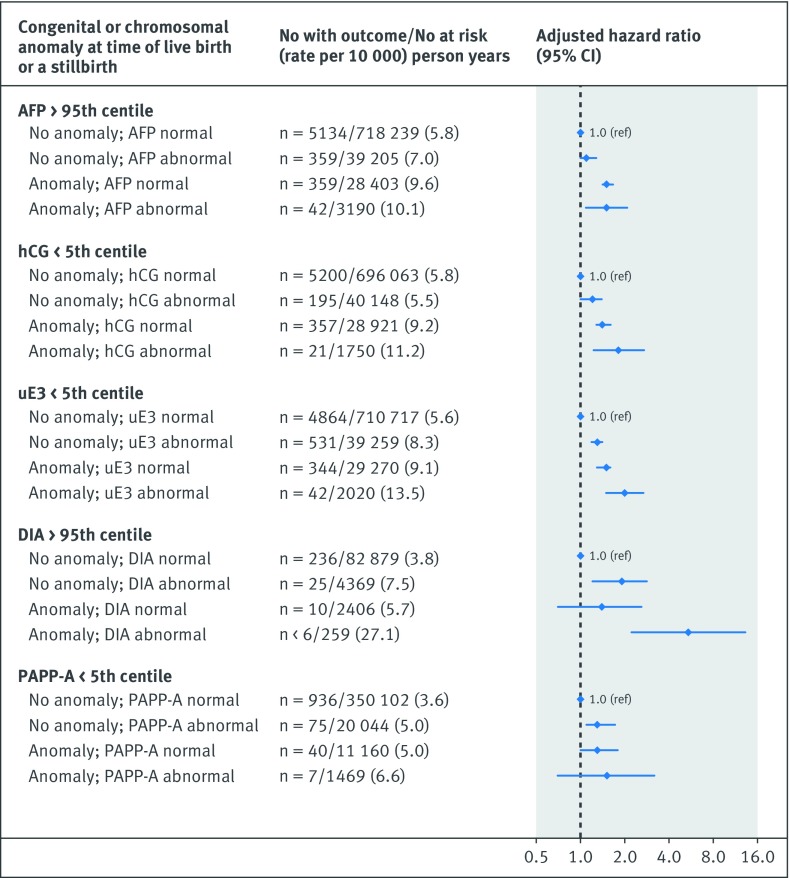
Evaluation of each biochemical analyte in absence or co-presence of recognised chromosomal or congenital anomaly (limited to live births or stillbirths). Referent was normal biochemical measure in conjunction with absence of perinatal or maternal factor. Adjusted for maternal age, gravidity, fifth of neighbourhood income, rural residence, and gestational age, each at the time of prenatal biochemical screening, as well as maternal diabetes mellitus, chronic hypertension, renal disease, tobacco/illicit drug use, and dyslipidaemia within 365 days preceding the start of the index pregnancy, up to and including 365 days after the start of the index pregnancy

**Fig 2:**
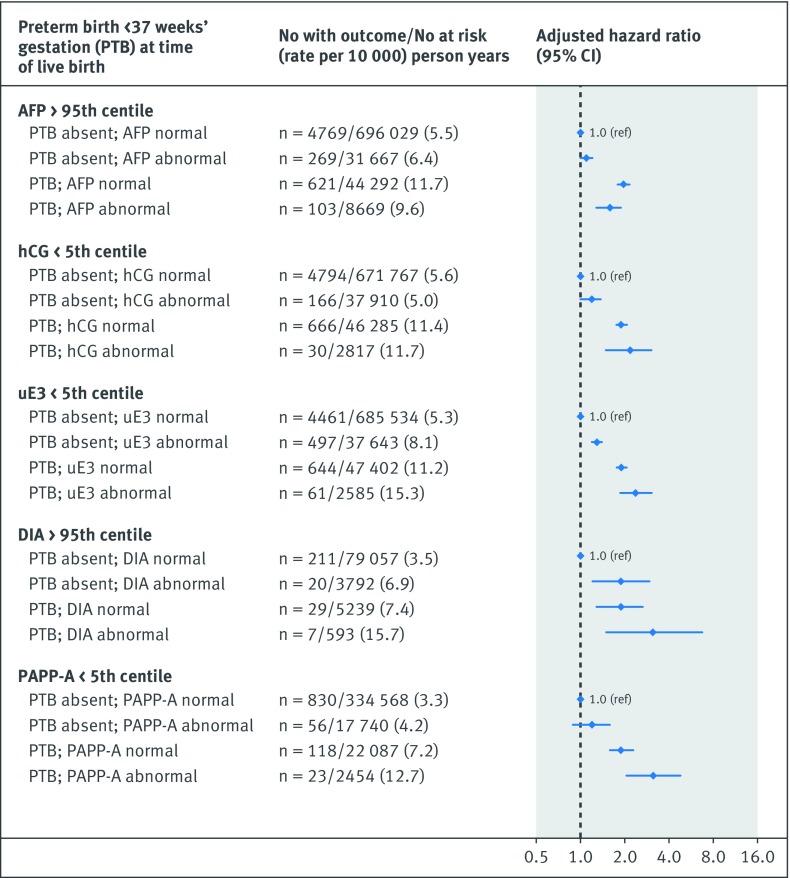
Evaluation of each biochemical analyte in absence or co-presence of preterm birth before 37 weeks’ gestation (limited to live births). Referent was normal biochemical measure in conjunction with absence of perinatal or maternal factor. Adjusted for maternal age, gravidity, fifth of neighbourhood income, rural residence, and gestational age, each at time of prenatal biochemical screening, as well as maternal diabetes mellitus, chronic hypertension, renal disease, tobacco/illicit drug use, and dyslipidaemia within 365 days preceding start of index pregnancy, up to and including 365 days after start of index pregnancy

**Fig 3:**
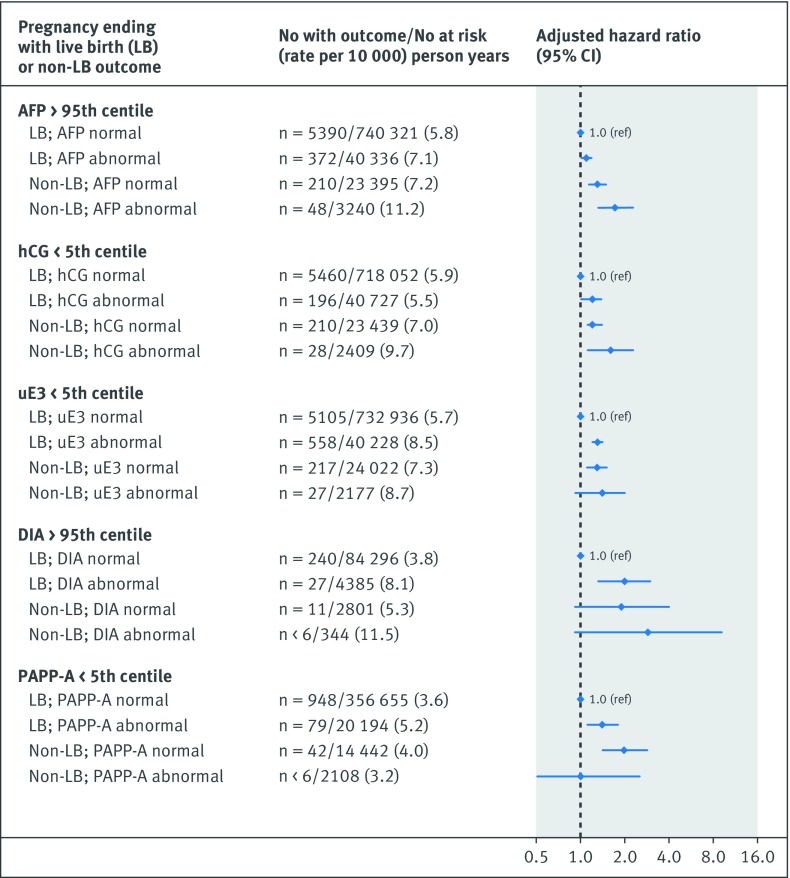
Evaluation of each biochemical analyte for non-live birth versus live birth (top). Referent was normal biochemical measure in conjunction with absence of perinatal or maternal factor. Adjusted for maternal age, gravidity, fifth of neighbourhood income, rural residence, and gestational age, each at time of prenatal biochemical screening, as well as maternal diabetes mellitus, chronic hypertension, renal disease, tobacco/illicit drug use, and dyslipidaemia within 365 days preceding start of index pregnancy, up to and including 365 days after start of index pregnancy

**Fig 4:**
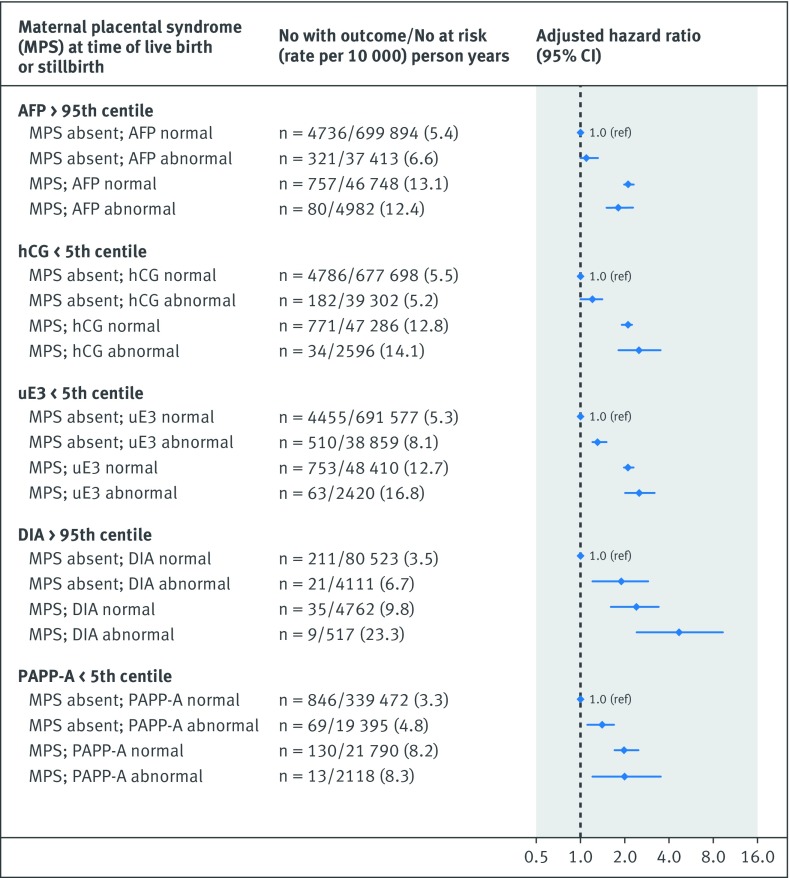
Evaluation of each biochemical analyte in absence or co-presence of maternal placental syndrome—pre-eclampsia, gestational hypertension, or placental abruption or infarction (limited to live births or stillbirths). Referent was normal biochemical measure in conjunction with absence of perinatal or maternal factor. Adjusted for maternal age, gravidity, fifth of neighbourhood income, rural residence, and gestational age, each at time of prenatal biochemical screening, as well as maternal diabetes mellitus, chronic hypertension, renal disease, tobacco/illicit drug use, and dyslipidaemia within 365 days preceding start of index pregnancy, up to and including 365 days after start of index pregnancy

**Fig 5:**
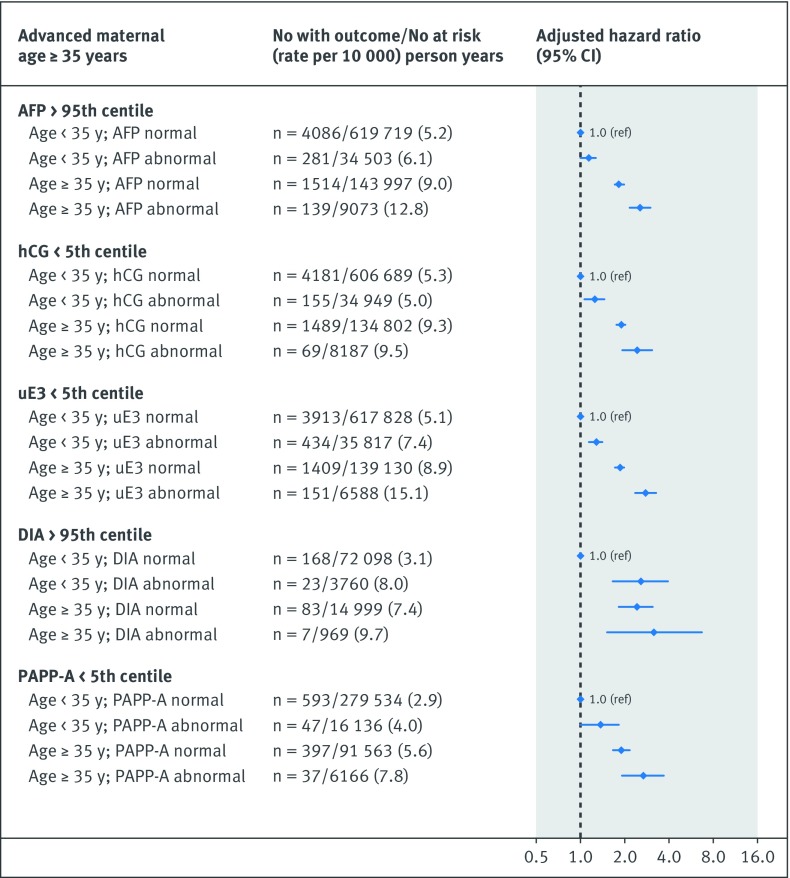
Evaluation of each biochemical analyte in absence or co-presence of maternal age ≥35 years at time of prenatal biochemical screening. Referent was normal biochemical measure in conjunction with absence of perinatal or maternal factor. Adjusted for gravidity, fifth of neighbourhood income, rural residence, and gestational age, each at time of prenatal biochemical screening, as well as maternal diabetes mellitus, chronic hypertension, renal disease, tobacco/illicit drug use, and dyslipidaemia within 365 days preceding start of index pregnancy, up to and including 365 days after start of index pregnancy

The risk of the primary cardiovascular disease composite outcome was notably higher in the co-presence of a recognised congenital or chromosomal anomaly at birth and either an abnormal unconjugated estriol (adjusted hazard ratio 2.0, 1.5 to 2.7) or dimeric inhibin-A (5.3, 2.2 to 13.0) (additional analysis 4; fig 1). Women with a preterm live birth were at significantly higher risk of cardiovascular disease than those with a term live birth, and the risk was highest in the co-presence of each abnormal analyte, although confidence limits overlapped with the exception of serum alphafetoprotein (additional analysis 4; fig 2). The hazard ratio for cardiovascular disease was only marginally higher for pregnancies resulting in non-live birth outcome and an abnormal biochemical measure (additional analysis 4; fig 3). In contrast, a live birth or stillbirth pregnancy affected by a maternal placental syndrome had a higher risk of cardiovascular disease, especially with a concomitantly abnormal dimeric inhibin-A (adjusted hazard ratio 4.7, 2.4 to 9.4) (additional analysis 4; fig 4). Finally, the hazard ratio for cardiovascular disease was higher in the co-presence of maternal age 35 years or above, especially with any abnormal analyte (additional analysis 4; fig 5).

Among all women who underwent prenatal biochemical screening, the percentage of those who had it done in two or more pregnancies was 24% for alphafetoprotein, human chorionic gonadotropin, or unconjugated estriol, 7% for dimeric inhibin-A, and 20% for pregnancy associated plasma protein A (supplementary file 9). Among those with biochemical screening in more than one pregnancy, repeatedly abnormal results ranged from 1% for alphafetoprotein to 1.6% for dimeric inhibin-A (supplementary file 9). However, the risk of the cardiovascular disease composite outcome was not altered in a model limited to repeat pregnancies, also adjusted for the number of previous abnormal prenatal screening results (supplementary file 10).

The non-screened and screened cohorts each contained 750 742 women (supplementary file 11). Comparing the non-screened and screened cohorts, we saw important standardised differences above 0.10 only for maternal age, rural residence, and gravidity. The primary cardiovascular disease composite outcome was only marginally more likely in the non-screened than the screened pregnancies (adjusted hazard ratio 1.1, 1.0 to 1.1) (additional analysis 6; supplementary file 11).

## Discussion

Women with abnormal prenatal biochemical screening results, especially those with an elevated serum dimeric inhibin-A, were found to be at modestly higher risk of a broad premature cardiovascular disease composite outcome, largely arising before age 50 years. The hazard for the secondary outcome of major adverse cardiovascular events was particularly higher in women with an elevated serum dimeric inhibin-A or low pregnancy associated plasma protein A. The risk of cardiovascular disease was more pronounced as the number of abnormal screening analytes increased, particularly in the co-presence of a newborn congenital anomaly, preterm birth, a maternal placental syndrome, or advanced maternal age.

### Limitations and strengths of study

Of all recognised pregnancies, about 49% had prenatal biochemical screening offered in a universal free healthcare setting. This apparently low rate may be partly explained by our inclusion of some pregnancies that ended before 20 weeks’ gestation. Women who had screening differed minimally at baseline from those who did not, and their respective incidence rates of cardiovascular disease were similar (supplementary file 11).

We randomly selected one pregnancy per woman, which should have produced an unbiased estimate of her risk of cardiovascular disease in relation to her prenatal screening test result, even though fairly consistent between pregnancy correlations in serum markers have been reported previously.^19^ ^20^ Adjustment for number of previous pregnancies with abnormal prenatal screening results did not change our findings. The study lacked direct data about the relation between each prenatal biochemical analyte and its prediction of a fetal anomaly, but we did further analyse the risk of cardiovascular disease by the co-presence or absence of an anomaly at birth, assuming that the related diagnostic codes were accurate. Some screened pregnancies may have ended in a miscarriage or an induced abortion missed by the administrative datasets. Not all analytes showed a direct relation with cardiovascular disease (supplementary file 3), and although we derived the abnormal multiple of the median cut-off point for each by using the best possible approach, that method has not been previously validated. At a more discrete cut-off point, such as the 1st or 99th centile, the main findings were marginally increased, however (supplementary file 8).

The cardiovascular disease outcomes were confined to hospital admissions arising well after pregnancy, and many of the core diagnostic codes have been shown to be valid and accurate (see final column in supplementary file 2). Although some out-of-hospital cardiovascular disease events leading to death would be missed, the secondary outcome of major adverse cardiovascular events included fatalities. Potential confounders between an abnormal analyte and the risk of cardiovascular disease, including diabetes mellitus, renal disease, chronic hypertension, dyslipidaemia, and ethnicity,^21^ ^22^ ^23^ were each accounted for in the models. Nevertheless, about 10% of pregnancies lacked information on maternal weight, and height and menopausal status were entirely unknown. Certainly, the relation between an abnormal analyte and maternal cardiovascular disease risk can be explained by a series of factors (supplementary file 1).

### Other studies

No previous data exist about the use of prenatal biochemical screening to estimate the long term cardiovascular health of a woman. In non-pregnant adults, a high serum pregnancy associated plasma protein A has been evaluated in relation to plaque instability in acute coronary syndrome, but its clinical utility remains uncertain.^24^ Although some understanding of the functional effects of dimeric inhibin-A in human reproduction and pregnancy exists, its role in cardiovascular disease is largely unknown.^25^ Hence, whether one or more of the abnormal analytes evaluated in this study are merely reflective of placental vascular disease or are persistently abnormal outside of pregnancy as a reflection of, or contributor to, vascular injury remains to be determined.^3^

### Clinical and policy relevance

To date, prenatal biochemical screening has focused on fetal screening and, perhaps, placenta related pregnancy outcomes.^2^ ^10^ ^14^ A practical question raised by this study is which prenatal biochemical analyte, or combination of analytes, is predictive of future cardiovascular disease. Furthermore, given that the ratio of serum soluble fms-like tyrosine kinase 1 to placental growth factor has recently been validated as a biochemical predictor of pre-eclampsia,^26^ it too can be evaluated as a marker of persistent endothelial dysfunction and risk of cardiovascular disease after pregnancy.^27^ ^28^ Regardless, we need better data about whether prenatal biochemical screening offers additive information over that provided by conventional cardiovascular disease risk factors and adverse events in pregnancy,^4^ ^5^ ^6^ ^7^ ^8^ such as the maternal placental syndromes or preterm delivery. One approach might be an analysis of risk reclassification, assessing the proportion of women whose level of risk changes if the values of prenatal screening are added to a list of conventional risk factors.

International guidelines for the prevention of stroke and cardiovascular disease now recommend screening for cardiovascular risk factors in women with previous maternal placental syndromes, such as pre-eclampsia.^29^ ^30^ However, maternal recall of a hypertensive disorder in a previous pregnancy lacks sensitivity.^31^ Tens of millions of women worldwide have completed prenatal biochemical screening, and although the original intent was to screen for certain congenital and chromosomal anomalies, a massive amount of data now exists that might be applied to better estimate a woman’s long term risk of cardiovascular disease. This need not only be done prospectively. In the age of data mining and machine learning,^32^ identifying a woman who previously had abnormal prenatal biochemical screening and conveying that information about her higher cardiovascular disease risk to her or her healthcare provider would seem possible. However, before giving consideration to these points, our findings should be replicated in other populations of women who have undergone prenatal biochemical screening, including an evaluation of various combinations of analytes.

**What is already known on this topic**

Prenatal biochemical screening for trisomies and birth defects has been completed among millions of women
Abnormal prenatal biochemical screening is related to a higher risk of pre-eclampsia, and pre-eclampsia is linked to premature cardiovascular disease in women
No previous study seems to have examined the risk of maternal cardiovascular disease, or any subtype of cardiovascular disease, in relation to prenatal biochemical screening

**What this study adds**

Women with abnormal prenatal biochemical screening results were at modestly higher risk of a broad premature cardiovascular disease composite outcome, as well as the secondary outcome of major adverse cardiovascular events
A massive amount of data now exists that might be applied to better estimate a woman’s long term risk of cardiovascular disease

## Web Extra

Extra material supplied by the author

## Notes

Contributors: JGR and ALP developed the study concept, analysed and interpreted the data, drafted and revised the manuscript, and approved the final version. TH and WM interpreted the data, revised the manuscript, and approved the final version. EC revised the manuscript and approved the final version. All authors had full access to all of the data in the study and can take responsibility for the integrity of the data and the accuracy of the data analysis. The corresponding author attests that all listed authors meet authorship criteria and that no others meeting the criteria have been omitted. JGR is the guarantor

Ethical approval: The Research Ethics Boards of the Sunnybrook Health Sciences Centre and the North York General Hospital granted ethics approval.

Transparency declaration: The lead author affirms that the manuscript is an honest, accurate, and transparent account of the study being reported; that no important aspects of the study have been omitted; and that any discrepancies from the study as originally planned (and, if relevant, registered) have been explained.

Data sharing: No additional data available. Only ALP is permitted to access the data.
