# Supplementary material for: Prenatal biochemical screening and long term risk of maternal cardiovascular disease: population based cohort study
Source: BMJ. 2018 Jul 11;362:k2739. doi: 10.1136/bmj.k2739 (PMC6039827; doi:10.1136/bmj.k2739)
Supplement: Supplementary file 1 — Supplementary files [file rayj043743.ww1.pdf]

Supplementary file 1. Conceptual framework. The main study exposure is an abnormal prenatal biochemical screening analyte (alphafetoprotein [AFP], total human chorionic gonadotropin [hCG], unconjugated estriol [uE3], dimeric inhibin-A [DIA] and pregnancy-associated plasma protein A [PAPP-A]) The main study outcome is cardiovascular disease. Potential confounders and mediators of the relation between placental vascular disease or an abnormal prenatal biochemical screening analyte and subsequent cardiovascular disease are also shown.

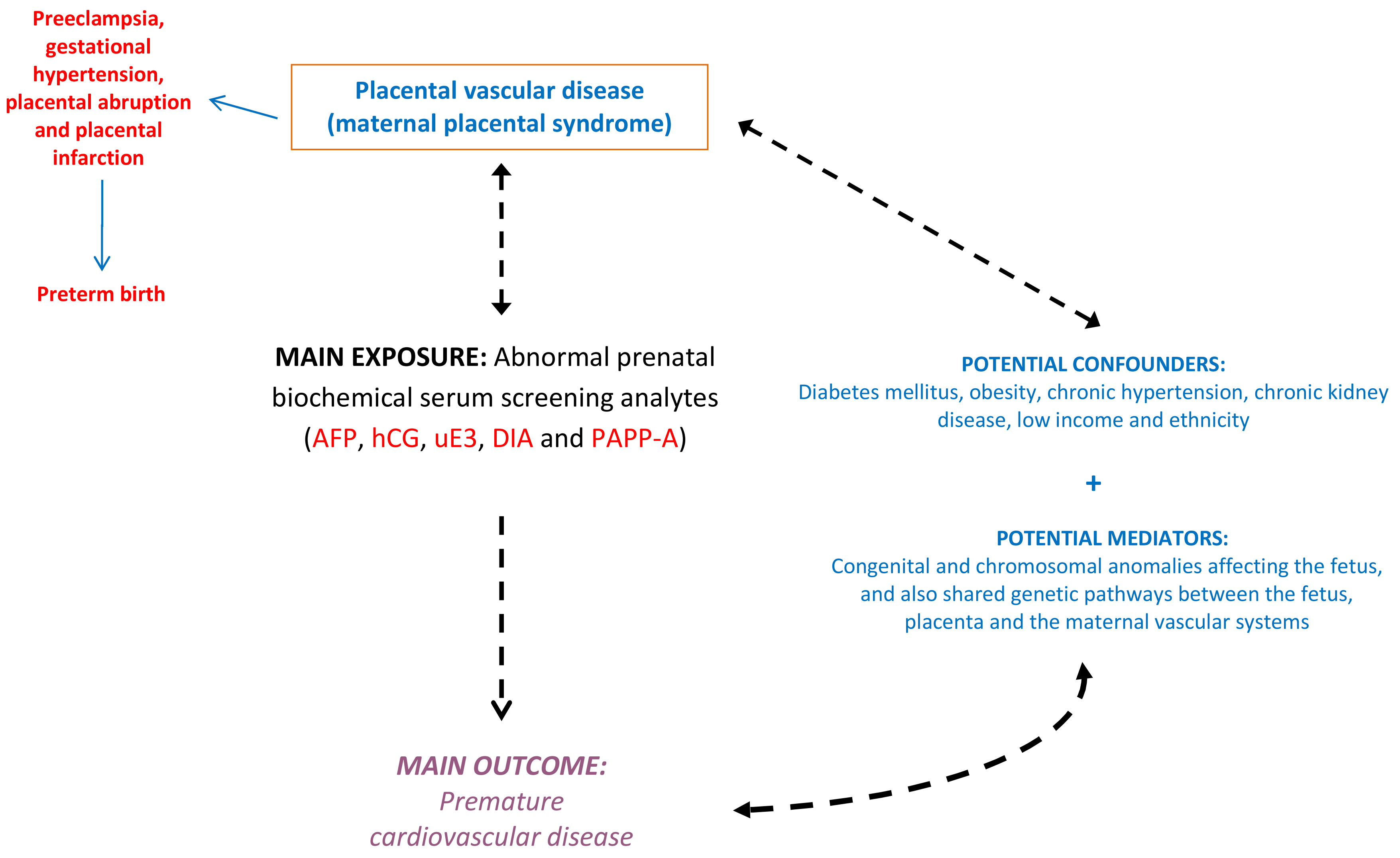

Supplementary file 2. Variables used to define cohort entry and exclusion criteria, study exposures, outcomes and adjustment variables.

Assessment	Timing	Disease or procedure or condition	ICD-9 [ICD-10-CA] codes	OHIP ICD-9 diagnostic codes or fee codes {or other source if in parentheses}	PubMed link to related validation studies for some codes
Cohort entry criterion	May 20, 1993 to December 29, 2011	Women aged 12 to 55 who underwent maternal serum screening from 11 to 20 weeks' gestation	--	{Ontario Maternal Serum Screening (OMSS) database}	
Exclusion criteria	Within ≤ 5 years before the maternal serum screening date in the index pregnancy	Coronary artery disease	410, 411, 413, 414.0, 429.2 [I20, I21, I24, I25.0, I25.1, I51.3] 48* [1HZ80*, 1IJ50*, 1IJ55*, 1IJ57*, 1IJ76*, 1IJ80*, 1IK80*, 1IK87*, 1IL35*, 2IL70*, 3IP10*]	410, 412, 413, 429	
	Same	Cardiac dysrhythmia	427.3 [I48], 427.1 [I47.2], 427.4 [I49.0], 427.2 [I47.2]	427	
	Same	Heart failure	428 [I50]	428	
	Same	Pericardial disease, endocarditis, myocarditis, cardiomyopathy or peripartum cardiomyopathy, valvular heart disease	420-425 [I30-I43], 674.5 [O90.3], 390-392 [I00-I02, I05-I09]	398	
	Same	Congenital heart disease	745-746, 7470, 7471, 7472, 7473, 7474 [Q20-Q26]	745-747	
	Same	Cerebrovascular disease	433, 434, 436, 437.0, 437.1, 437.8, 437.9 [G46, I63.0-I66.9, I67.2, I67.8] 50.11*, 50.12* [1JE57*, 1JW57*, 1JX57*]	432, 436, 437	

Assessment	Timing	Disease or procedure or condition	ICD-9 [ICD-10-CA] codes	OHIP ICD-9 diagnostic codes or fee codes {or other source if in parentheses}	PubMed link to related validation studies for some codes
	Same	Peripheral arterial disease	440.0, 440.2, 444 [I70.0, I70.2, I74], 50.18*, 50.28*, 50.38*, 51.24*, 51.25*, 51.26*, 51.29* [1JM76*, 1JW76*, 1JX76*, 1KA76*, 1KE76*, 1KG57*, 1KR76*, 1KR87LA*, 1KT76*, 1ID76MU*, 1KG76*, 1KG87*]	443	
	At the time of the maternal serum screening	Non-Ontario resident or invalid OHIP number	--	{Registered Persons Database (RPDB) contains demographic information and encrypted healthcare numbers for all individuals eligible for OHIP}	
	Same	Maternal serum screening results missing, duplicated or outside of 11^{+0}-13^{+6} weeks (pregnancy associated plasma protein A) or 15^{+0}-20^{+6} weeks (all other analytes)	--	{OMSS}	
`	Same	Date of last contact was prior to or on the date of the maternal serum screening	--	{RPDB}	
	Same	Implausibly low total human chorionic gonadotropin ≤ 0.1st percentile Multiple of the Median (MoM)	--	{OMSS}	

Assessment	Timing	Disease or procedure or condition	ICD-9 [ICD-10-CA] codes	OHIP ICD-9 diagnostic codes or fee codes {or other source if in parentheses}	PubMed link to related validation studies for some codes
Main study exposures	At the time of the maternal serum screening	Abnormal serum alphafetoprotein (AFP)	--	{OMSS}	
	Same	Abnormal serum total human chorionic gonadotropin (hCG)	--	{OMSS}	
	Same	Abnormal serum unconjugated estriol (uE3)	--	{OMSS}	
	Same	Abnormal serum dimeric inhibin-A (DIA)	--	{OMSS}	
	Same	Abnormal serum pregnancy-associated plasma protein A (PAPP-A)	--	{OMSS}	

Assessment	Timing	Disease or procedure or condition	ICD-9 [ICD-10-CA] codes	OHIP ICD-9 diagnostic codes or fee codes {or other source if in parentheses}	PubMed link to related validation studies for some codes
Study outcomes	Starting at 365 days after the start of the index pregnancy (January 27, 1994 to March 31, 2016) = "t_0"	#1: Cardiovascular disease composite of any hospitalization or revascularization for coronary artery, cerebrovascular or peripheral arterial disease, or any hospitalization for heart failure or dysrhythmia. Censored on death or at the end of follow-up.	Coronary artery disease:<br>410, 411, 413, 414.0, 429.2, [I20, I21, I24, I25.0, I25.1, I51.3], 48* [1HZ80*, 1IJ50*, 1IJ55*, 1IJ57*, 1IJ76*, 1IJ80*, 1IK80*, 1IK87*, 1IL35*, 2IL70*, 3IP10*]<br><br>Cerebrovascular disease:<br>431, 433, 434, 436, 437.0, 437.1 [G46, I61, I63.0-I66.9, I67.2, I67.8], 50.11*, 50.12* [1JE57*, 1JW57*, 1JX57*]<br><br>Peripheral arterial disease:<br>440.0, 440.2, 444 [I70.0, I70.2, I74], 50.18*, 50.28*, 50.38*, 51.24*, 51.25*, 51.26*, 51.29* [1JM76*, 1JW76*, 1JX76*, 1KA76*, 1KE76*, 1KG57*, 1KR76*, 1KR87LA*, 1KT76*, 1ID76MU, 1KG76*, 1KG87*]<br><br>Heart failure: 428 [I50]<br><br>Cardiac dysrhythmia:<br>427.3 [I48], 427.1 [I47.2], 427.4 [I49.0], 427.2 [I47.9]	--	https://www.ncbi.nlm.nih.gov/pubmed/12177647<br><br>https://www.ncbi.nlm.nih.gov/pubmed/27426016<br><br>https://www.ncbi.nlm.nih.gov/pubmed/12177647<br><br>https://www.ncbi.nlm.nih.gov/pubmed/12177647

Assessment	Timing	Disease or procedure or condition	ICD-9 [ICD-10-CA] codes	OHIP ICD-9 diagnostic codes or fee codes {or other source if in parentheses}	PubMed link to related validation studies for some codes
	Same	#2: Major Adverse Cardiovascular Events (MACE) composite outcome of myocardial infarction, stroke or death. Censored on end of follow-up only.	Myocardial infarction: 410 [I21]<br><br>Stroke:<br>431, 433, 434 [I61, I63, I64]	{Death in RPDB}	
Covariates	At the time of the maternal serum screening	Maternal age	--	{OMSS}	
	Same	Maternal area-level income quintile	--	{Statistics Canada census data}	
	Same	Maternal rural residence	--	{Statistics Canada census data}	
	Same	Maternal ethnicity	--	{OMSS}	
	At time of the maternal serum screening in the index pregnancy	Maternal weight (kg)	--	{OMSS}	
	At the time of the maternal serum screening (if available), otherwise calculated from all pregnancies in	Gravidity	Calculated based on all pregnancy outcomes in DAD, SDS, NACRS and OHIP	{OMSS}	

Assessment	Timing	Disease or procedure or condition	ICD-9 [ICD-10-CA] codes	OHIP ICD-9 diagnostic codes or fee codes {or other source if in parentheses}	PubMed link to related validation studies for some codes
	Up to 2 years before "t_0" (i.e. 365 days before the start of the index pregnancy, up to and including 365 days after the start of the index pregnancy)	Diabetes mellitus	250, 648.8 [E10, E11, E13, E14, O244]	250 or {OMSS}	https://www.ncbi.nlm.nih.gov/pubmed/11874939
	Same	Chronic hypertension	401, 405, 642.0-642.2, 642.7 [I10, I15, O10, O11]	401	https://www.ncbi.nlm.nih.gov/pubmed/19858407
	Same	Dyslipidemia	272.0, 272.1, 272.3, 272.4, 272.5 [E78]	272	
	Same	Renal disease	584.5-584.9, 669.3, 958.5 634.3, 635.3, 636.3, 637.3, 638.3, 639.3, 250.4x, 274.1x, 403.xx, 404.xx, 405.01, 405.11, 405.91, 440.1, 446.21, 581.xx, 582.xx, 583.xx, 585.x, 586, 587.x, 588.0, 588.8x, 588.9, 590.0x, 593.7x, 791.0, 794.4 [N17.x, O08.4, T79.5, O90.4, E10.20, E10.21, E10.23, E11.20, E11.21, E11.23, M10.39, I12, I13, I15.0, I70.1, M31.0, N01.x, N03.x, N04.x, N05.x, N06.x, N07.x, N08.x, N11.x, N12, N13.7, N13.8, N13.9, N14.x, N15.x, N16.x, N18.x, N19.x, N25.0, N25.8, N25.9, N26, R80, R94.4]	403, 581, 585	https://www.ncbi.nlm.nih.gov/pubmed/23560464

Assessment	Timing	Disease or procedure or condition	ICD-9 [ICD-10-CA] codes	OHIP ICD-9 diagnostic codes or fee codes {or other source if in parentheses}	PubMed link to related validation studies for some codes
	Same	Drug dependence or tobacco use	291, 292, 2940, 303, 304, 305, 648.3, 649.0, 6555, 980 [F10-F19, F55, G312, O354, O355, T51, T652, Z720, Z721, Z722]	291, 292, 303, 304, 305	
Censoring variables	Starting at 365 days after the start of the index pregnancy (January 27, 1994 to March 31, 2016) = "t_0"	Death (for study outcome #1 only)	--	{RPDB}	
	March 31, 2016	End of study	--	--	
Stratification variables	Determined by the end of the pregnancy	Livebirth	M_STILLBIRTH='F' in MOMBABY (see https://datadictionary.ices.on.ca/Applications/DataDictionary/Library.aspx?Library=MOMBABY)	--	
	Same	Stillbirth[a]	M_STILLBIRTH='T' in MOMBABY (see https://datadictionary.ices.on.ca/Applications/DataDictionary/Library.aspx?Library=MOMBABY)	--	
	Same	Induced abortion[a]	635 [O04, O08] AND 81.01*, 87.0*, 87.1*, 87.21*, 87.29* [5CA89*, 5CA88*, 5CA20FK*, 5CA24*] AND prsuff not in 8, 9	Fee Code: S785, A920, P001 and ICD-9: 635, 895; OR Fee Code: S752 and ICD-9: 635, 895	

Assessment	Timing	Disease or procedure or condition	ICD-9 [ICD-10-CA] codes	OHIP ICD-9 diagnostic codes or fee codes {or other source if in parentheses}	PubMed link to related validation studies for some codes
	Same	Spontaneous abortion (miscarriage) or ectopic pregnancy[a]	632, 633, 634 [O00, O021, O03]	Fee Codes: A920, P001 and ICD-9: 632, 633, 634, 640; OR Fee Code: A922; OR Fee Codes: S752, S785 and ICD-9: 632, 633, 634, 640; OR Fee Codes: S756, S768, S784, S770	
	Same	Unknown[a]	Pregnancy outcome not documented in the CIHI-DAD, SDS or NACRS.	Pregnancy outcome not documented in OHIP.	
Stratification variables	At the time of the index livebirth or stillbirth delivery hospitalization	Preeclampsia or eclampsia[b]	642.4-642.7 [O11, O14, O15]	--	https://www.ncbi.nlm.nih.gov/pubmed/19527567
	Same	Gestational hypertension[b]	642.3, 642.9 [O13]	--	
	Same	Placental abruption[b]	641.2 [O45]	--	
	Same	Placental infarction[b]	656.7 [O43.1, O43.801, O43.803, O43.809, O43.811, O43.813, O43.819, O43.9]	--	
	Same	Congenital or chromosomal anomaly	648.9, 655.0, 655.1, 74, 75 [O28, O35.0, O35.1, Q]	--	

Assessment	Timing	Disease or procedure or condition	ICD-9 [ICD-10-CA] codes	OHIP ICD-9 diagnostic codes or fee codes {or other source if in parentheses}	PubMed link to related validation studies for some codes
	At the time of the index livebirth delivery hospitalization	Preterm live birth < 37 completed weeks' gestation	Before FY2002: 644.2, 765 [O60, P07.2, P07.3]<br>FY2002 onward: M_GESTWKS_DEL or B_GESTWKS_DEL < 37 in MOMBABY (https://datadictionary.ices.on.ca/Applications/DataDictionary/Library.aspx?Library=MOMBABY)	--	

*Indicates coding by Canadian Classification of Procedures (corresponding to ICD-9 years) or Canadian Classification of Interventions (corresponding to ICD-10-CA years)

[a]Each is a pregnancy outcome used to broadly define non-livebirth.

[b]Each is a condition used to broadly define a Maternal Placental Syndrome (MPS).

CIHI: Canadian Institute for Health Information; DAD: Discharge Abstract Database; ICD-9: International Classification of Diseases, 9th Revision; ICD-10-CA: International Classification of Diseases, 10th Revision, Canada; NACRS: National Ambulatory Care Reporting System; OHIP: Ontario Health Insurance Plan; SDS: Same Day Surgery Database

Supplementary file 3. Fractional polynomial derived best fitting plots of the continuous relation between the multiple of the median (MoM) for serum alphafetoprotein (AFP) (*a*), unconjugated estriol (uE3) (*b*), total human chorionic gonadotropin (hCG) (*c*), pregnancy-associated plasma protein A (PAPP-A) (*d*) and dimeric inhibin-A (DIA) (*e*), and the respective hazard for the cardiovascular disease (CVD) composite outcome of any hospitalization or revascularization for coronary artery, cerebrovascular or peripheral arterial disease, heart failure or dysrhythmia. The 1st, 5th, 95th and 99th percentile cut-points for each analyte are shown by vertical dashed lines.

a

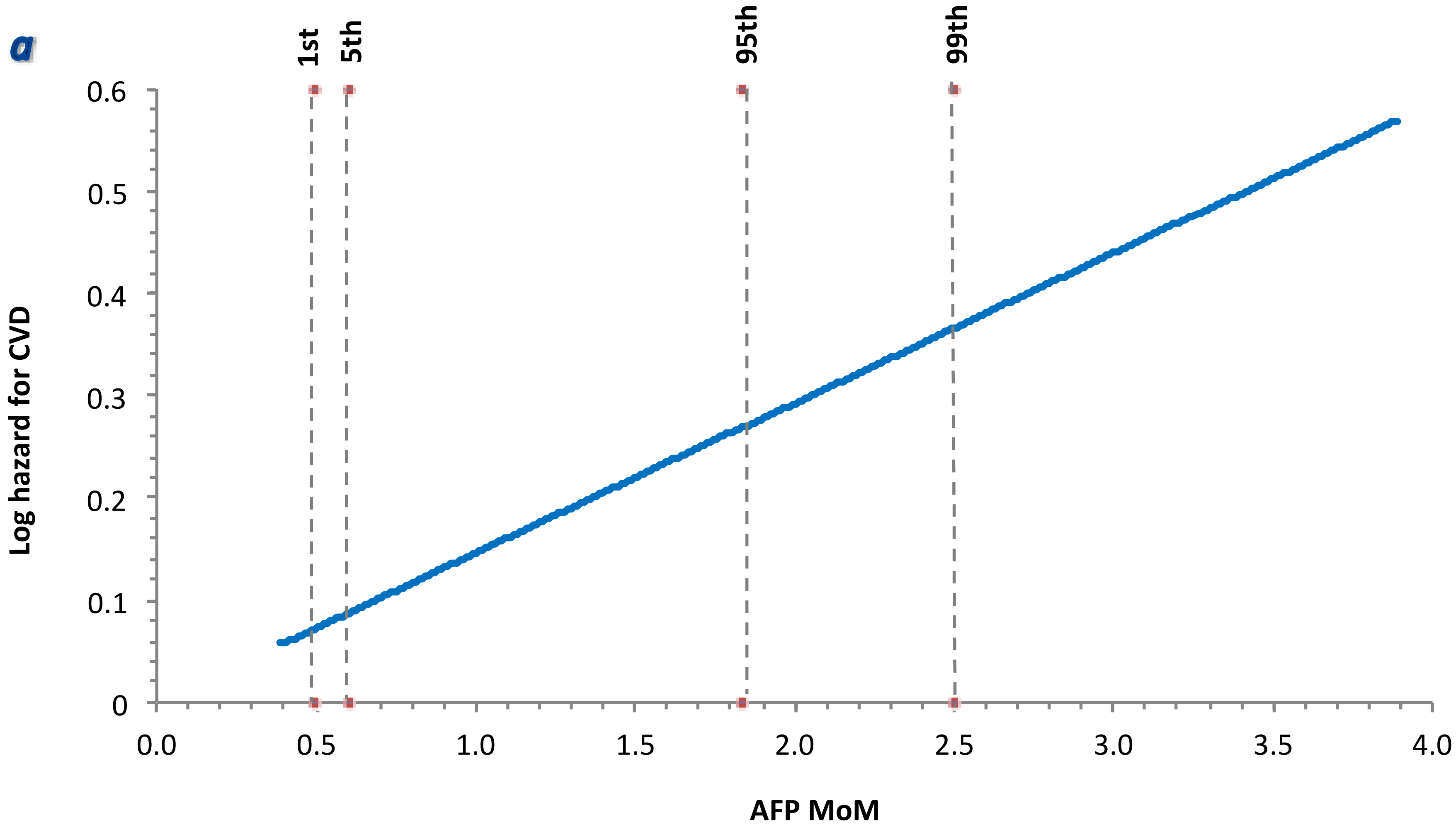

1st
5th
95th
99th
0.6
0.5
0.4
0.3
0.2
0.1
0
Log hazard for CVD
0.0
0.5
1.0
1.5
2.0
2.5
3.0
3.5
4.0
AFP MoM

b

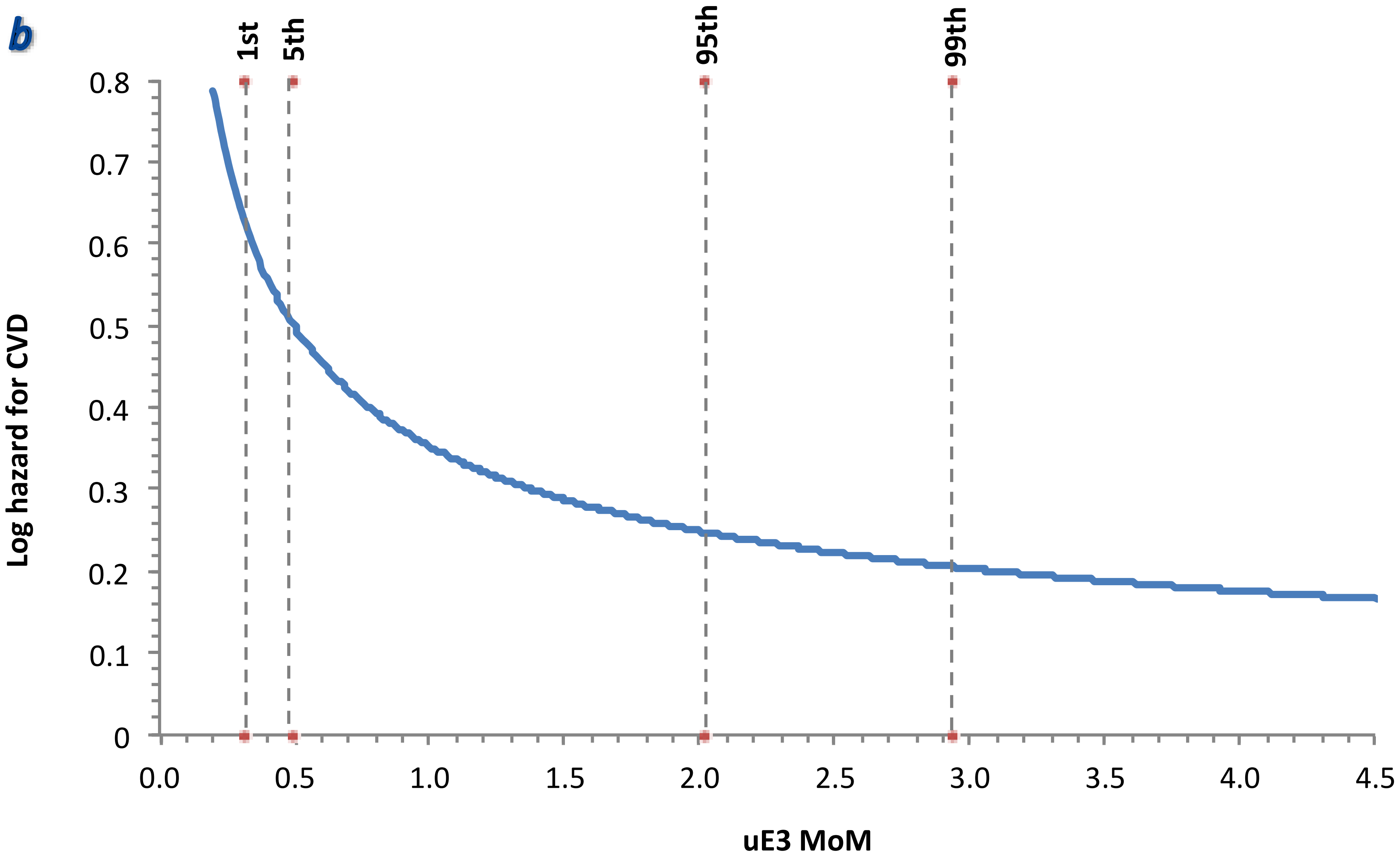
1st
5th
95th
99th
0.8
0.7
0.6
0.5
0.4
0.3
0.2
0.1
0
Log hazard for CVD
0.0
0.5
1.0
1.5
2.0
2.5
3.0
3.5
4.0
4.5
uE3 MoM

C

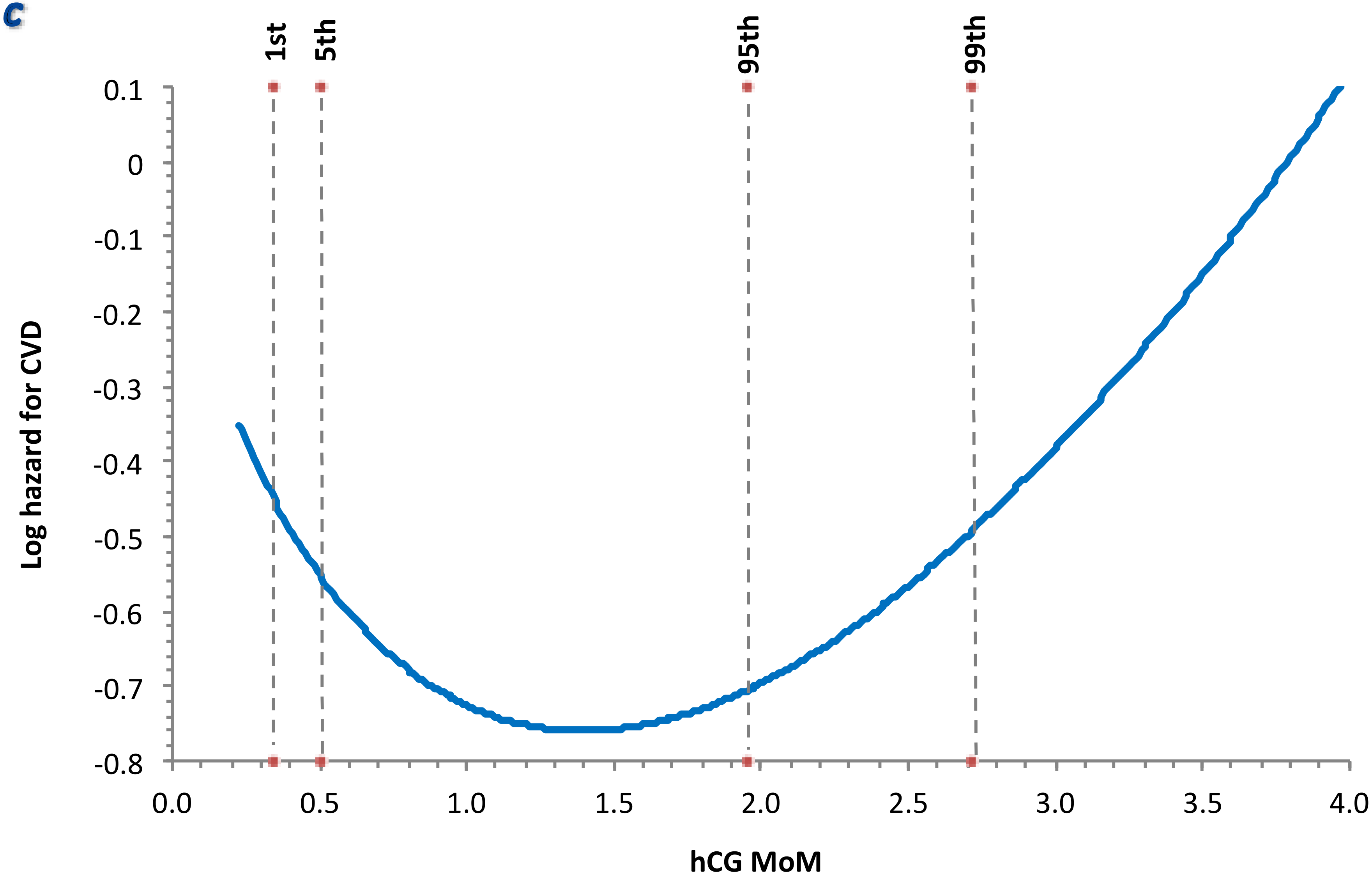
1st
5th
95th
99th
0.1
0
-0.1
-0.2
-0.3
-0.4
-0.5
-0.6
-0.7
-0.8
Log hazard for CVD
0.0
0.5
1.0
1.5
2.0
2.5
3.0
3.5
4.0
hCG MoM

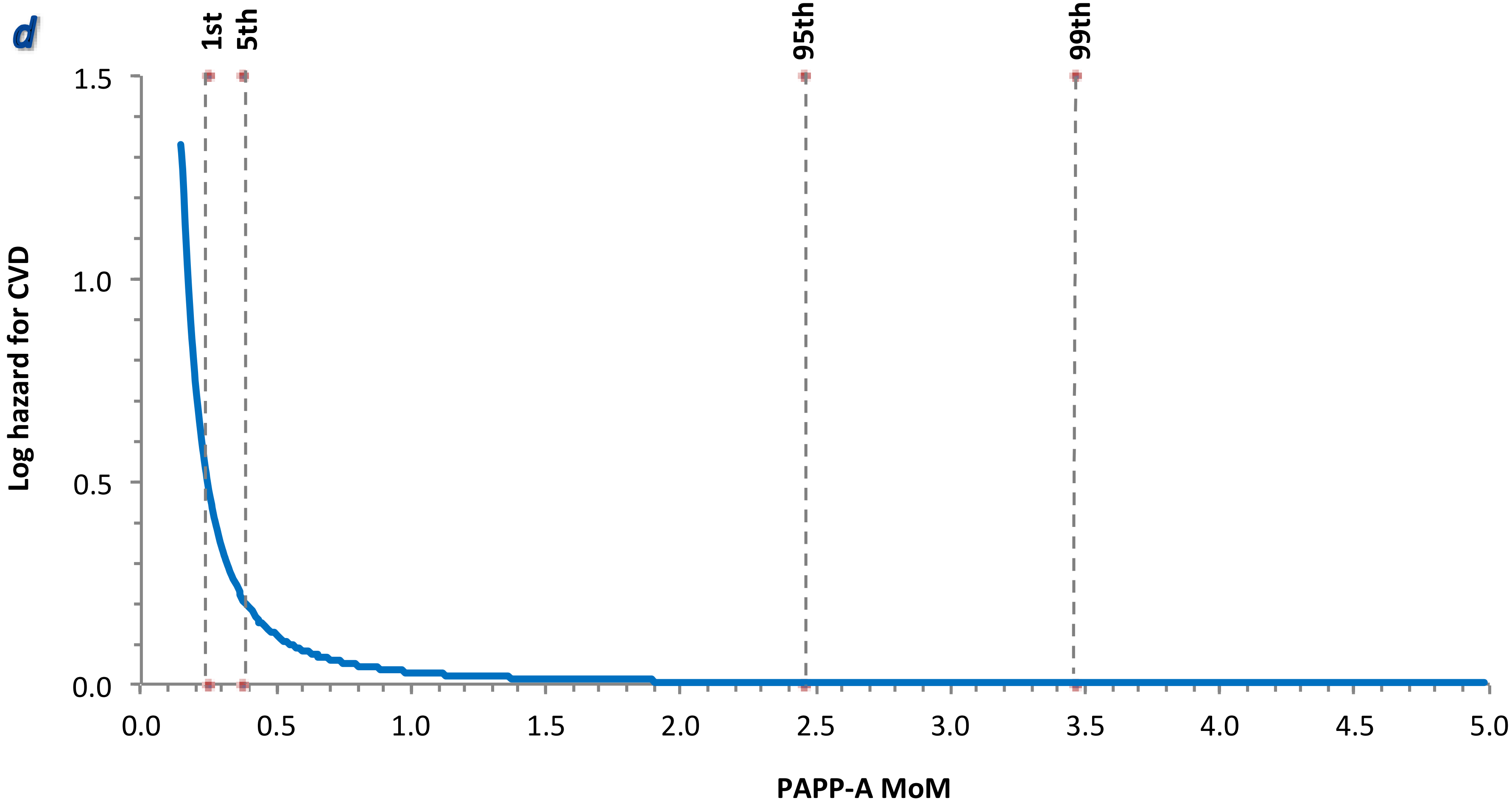
d
1st
5th
95th
99th
Log hazard for CVD
1.5
1.0
0.5
0.0
0.0
0.5
1.0
1.5
2.0
2.5
3.0
3.5
4.0
4.5
5.0
PAPP-A MoM

e

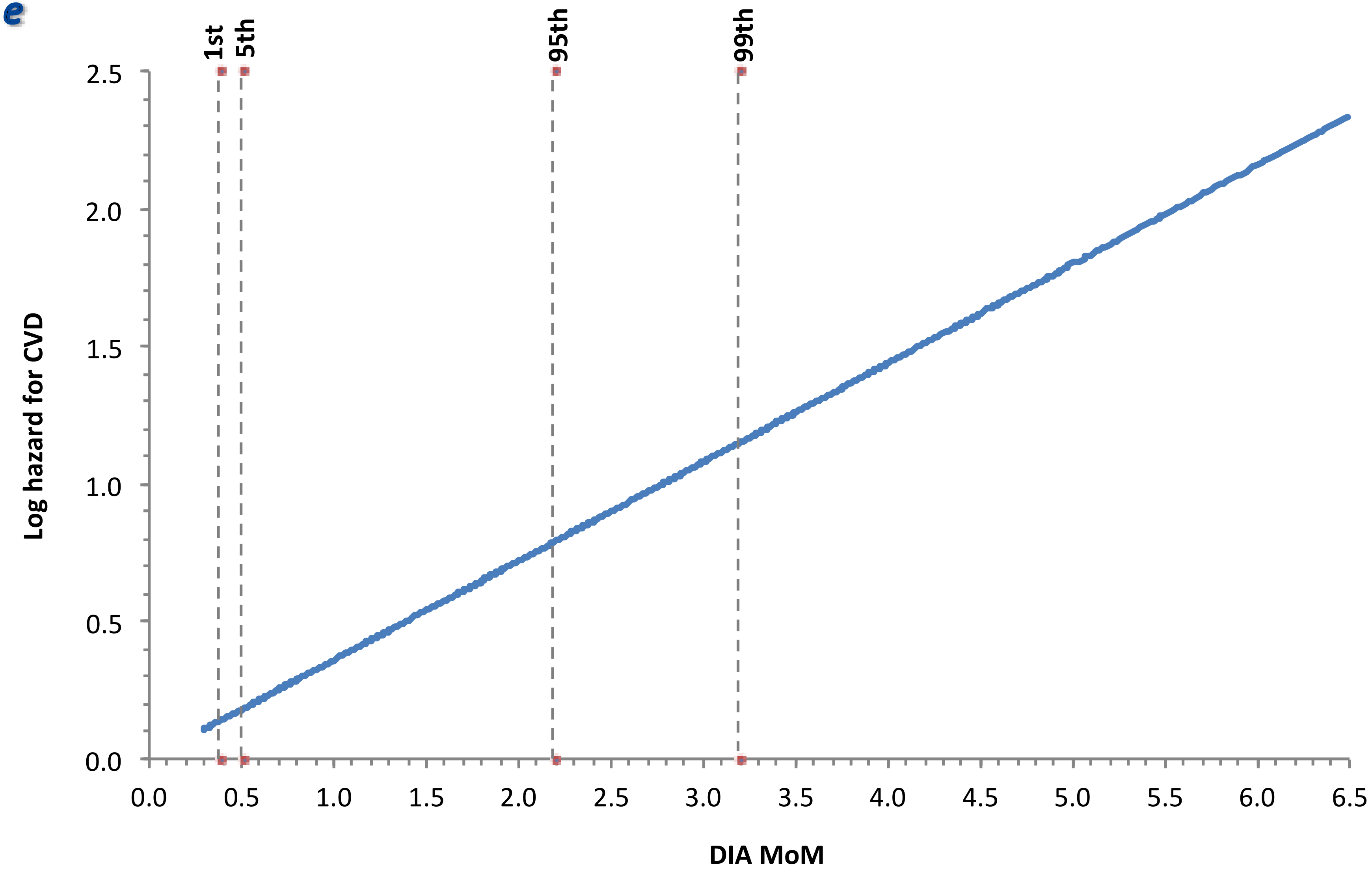
1st
5th
95th
99th
Log hazard for CVD
0.0
0.5
1.0
1.5
2.0
2.5
0.0
0.5
1.0
1.5
2.0
2.5
3.0
3.5
4.0
4.5
5.0
5.5
6.0
6.5
DIA MoM

Supplementary file 4. Flow chart of inclusion and exclusions

1 380 840 Pregnancies in the Ontario Maternal Serum Screening Database (May 20, 1993 to December 29, 2011)

171 150 Pregnancies excluded:

- **5153** Invalid maternal health card number
- **3481** Non-Ontario resident at the time of the specimen
- **1513** Specimen date was unreported or earlier than May 20, 1993
- **89 789** Specimen results were unreported, duplicated, invalid in gestational age, and/or consecutive specimens were incompatible in gestational age and date
- **535** Date of last contact preceded the specimen date
- **35 816** Coronary artery disease diagnosed ≤ 5 years before the specimen date
- **3275** Cerebrovascular disease diagnosed ≤ 5 years before the specimen date
- **3355** Peripheral artery disease diagnosed ≤ 5 years before the specimen date
- **20 844** Cardiac dysrhythmia diagnosed ≤ 5 years before the specimen date
- **1257** Heart failure diagnosed ≤ 5 years before the specimen date
- **1863** Pericardial disease, endocarditis, myocarditis, cardiomyopathy or peripartum cardiomyopathy, valvular heart disease diagnosed ≤ 5 years before the specimen date
- **3040** Congenital heart disease diagnosed ≤ 5 years before the specimen date
- **1229** Total human chorionic gonadotropin ≤ 0.1st percentile Multiple of the Median

1 210 146 Pregnancies included with at least one of the following biochemical serum screening analytes:

- **1 120 363** Had alphafetoprotein
- **1 110 624** Had unconjugated estriol
- **1 087 933** Had total human chorionic gonadotropin
- **499 790** Had pregnancy-associated plasma protein A
- **98 160** Had dimeric inhibin-A

855 536 Randomly selected pregnancies (one per woman) included with at least one biochemical serum screening analyte:

- **807 292** Had alphafetoprotein
- **799 363** Had unconjugated estriol
- **784 627** Had total human chorionic gonadotropin
- **393 399** Had pregnancy-associated plasma protein A
- **91 826** Had dimeric inhibin-A

Supplementary file 5 (Additional analysis 1). Risk of the primary cardiovascular disease composite outcome of any hospitalization or revascularization for coronary artery, cerebrovascular or peripheral arterial disease, heart failure or dysrhythmia, arising ≥ 365 days after the start of the index pregnancy, in association with an abnormal cut-point of the 5th or 95th percentile of the multiple of the median (MoM) for a given serum analyte. This analysis further adjusts for **maternal weight** at the time of prenatal biochemical screening, in a sub-set of all pregnancies.

		Cardiovascular disease composite outcome		
**Abnormal serum analyte**	**Cut-points used to define normal and abnormal**	**No. (incidence rate per 10000 person years)**	**Unadjusted hazard ratio (95% CI)**	**Adjusted hazard ratio (95% CI)[a]**
High alphafetoprotein	**Normal:** ≤ 95th percentile MoM (n = 688,426)	5,281 (5.9)	1.0 (Ref.)	1.0 (Ref.)
	**Abnormal**: > 95th percentile MoM (n = 39,317)	404 (7.6)	1.2 (1.1 to 1.3)	1.1 (1.0 to 1.3)
Low beta human chorionic gonadotropin	**Normal**: ≥ 5th percentile MoM (n = 673,615)	5,408 (6.0)	1.0 (Ref.)	1.0 (Ref.)
	**Abnormal**: < 5th percentile MoM (n = 36,205)	197 (5.8)	1.3 (1.2 to 1.4)	1.2 (1.0 to 1.4)
Low unconjugated estriol	**Normal**: ≥ 5th percentile MoM (n = 683,733)	5,055 (5.8)	1.0 (Ref.)	1.0 (Ref.)
	**Abnormal**: < 5th percentile MoM (n = 40,674)	553 (8.3)	1.3 (1.1 to 1.5)	1.3 (1.2 to 1.4)
High dimeric inhibin-A	**Normal**: ≤ 95th percentile MoM (n = 75,316)	217 (3.7)	1.0 (Ref.)	1.0 (Ref.)
	**Abnormal**: > 95th percentile MoM (n = 4147)	29 (8.9)	2.2 (1.5 to 3.2)	2.3 (1.5 to 3.4)
Low pregnancy-associated plasma protein A	**Normal**: ≥ 5th percentile MoM (n = 309,693)	861 (3.6)	1.0 (Ref.)	1.0 (Ref.)
	**Abnormal**: < 5th percentile MoM (n = 17,760)	67 (4.9)	1.4 (1.1 to 1.8)	1.3 (1.0 to 1.6)

[a] Adjusted for maternal weight (continuous), age (continuous), gravidity (1, 2+, missing), neighborhood income quintile (1, 2, 3, 4, 5, missing), rural residence (rural, urban, missing), ethnicity (Asian, Black Caucasian, Hispanic, Oriental, other, missing) and gestational age (continuous) – each at the time of prenatal biochemical screening – as well as maternal diabetes mellitus, chronic hypertension, renal disease, tobacco/drug use and dyslipidemia within 365 days preceding the start of the index pregnancy, up to and including 365 days after the start of the index pregnancy (i.e. time zero). Censored on death or arrival at the end of study date of March 31, 2016.

Supplementary file 6. Risk of the primary cardiovascular disease composite outcome of any hospitalization or revascularization for coronary artery, cerebrovascular or peripheral arterial disease, heart failure or dysrhythmia, arising ≥ 365 days after the start of the index pregnancy, in association with an abnormal cut-point of the 5th or 95th percentile of the multiple of the median (MoM) for a given serum analyte. This analysis further excludes women with pre-existing renal disease, chronic hypertension or dyslipidemia within 365 days preceding the start of the index pregnancy, and up to and including 365 days after the start of the index pregnancy (i.e., time zero).

		Cardiovascular disease composite outcome		
**Abnormal serum analyte**	**Cut-points used to define normal and abnormal**	**No. (incidence rate per 10000 person years)**	**Unadjusted hazard ratio (95% CI)**	**Adjusted hazard ratio (95% CI)[a]**
High alphafetoprotein	**Normal**: ≤ 95th percentile MoM (n = 728,734)	5206 (5.7)	1.0 (Ref.)	1.0 (Ref.)
	**Abnormal**: > 95th percentile MoM (n = 40,960)	381 (7.1)	1.2 (1.1 to 1.3)	1.2 (1.1 to 1.3)
Low beta human chorionic gonadotropin	**Normal**: ≥ 5th percentile MoM (n = 707,688)	5274 (5.8)	1.0 (Ref.)	1.0 (Ref.)
	**Abnormal**: < 5th percentile MoM (n = 40,825)	205 (5.6)	1.3 (1.1 to 1.5)	1.2 (1.1 to 1.4)
Low unconjugated estriol	**Normal**: ≥ 5th percentile MoM (n = 721,650)	4927 (5.6)	1.0 (Ref.)	1.0 (Ref.)
	**Abnormal**: < 5th percentile MoM (n = 40,836)	560 (8.4)	1.3 (1.2 to 1.5)	1.3 (1.2 to 1.4)
High dimeric inhibin-A	**Normal**: ≤ 95th percentile MoM (n = 82,345)	224 (3.6)	1.0 (Ref.)	1.0 (Ref.)
	**Abnormal**: > 95th percentile MoM (n = 4344)	24 (7.2)	2.0 (1.3 to 3.0)	1.9 (1.3 to 3.0)
Low pregnancy-associated plasma protein A	**Normal**: ≥ 5th percentile MoM (n = 350,425)	878 (3.4)	1.0 (Ref.)	1.0 (Ref.)
	**Abnormal**: < 5th percentile MoM (n = 20,660)	73 (4.7)	1.4 (1.1 to 1.8)	1.4 (1.1 to 1.7)

[a] Adjusted for maternal age (continuous), gravidity (1, 2+, missing), neighborhood income quintile (1, 2, 3, 4, 5, missing), rural residence (rural, urban, missing), ethnicity (Asian, Black Caucasian, Hispanic, Oriental, other, missing) and gestational age (continuous) – each at the time of prenatal biochemical screening – as well as maternal diabetes mellitus and tobacco/drug use within 365 days preceding the start of the index pregnancy, up to and including 365 days after the start of the index pregnancy (i.e., time zero). Censored on death or arrival at the end of study date of March 31, 2016.

Supplementary file 7. Breakdown of the sub-types of cardiovascular disease among the 6209 women who experienced a primary cardiovascular disease composite outcome event. Values do not sum to the total, as some women experienced more than one cardiovascular disease sub-type index event.

Cardiovascular disease sub-type	No. (%) of women
Coronary artery disease	3334 (53.7)
Cerebrovascular disease	1361 (21.9)
Peripheral arterial disease	1192 (5.9)
Heart failure	964 (15.5)
Dysrythmia	369 (19.2)

Supplementary file 8 (Additional analysis 3). Risk of the primary cardiovascular disease composite outcome of any hospitalization or revascularization for coronary artery, cerebrovascular or peripheral arterial disease, heart failure or dysrhythmia, arising ≥ 365 days after the start of the index pregnancy (upper), the coronary artery disease outcome arising ≥ 365 days after the start of the index (middle), and the cerebrovascular disease outcome arising ≥ 365 days after the start of the index (lower), each in association with an abnormal cut-point of the 1st or 99th percentile of the multiple of the median (MoM) for a given serum analyte.

		Cardiovascular disease composite outcome		
**Abnormal serum analyte**	**Cut-points used to define normal and abnormal**	**No. (incidence rate per 10,000 person years)**	**Unadjusted hazard ratio (95% CI)**	**Adjusted hazard ratio (95% CI)[a]**
High alphafetoprotein	Normal: ≤ 99th percentile MoM (n = 797,022)	5922 (6.0)	1.0 (Ref.)	1.0 (Ref.)
	Abnormal: > 99th percentile MoM (n = 10,270)	98 (7.3)	1.2 (1.0 to 1.4)	1.1 (0.9 to 1.3)
Low total human chorionic gonadotropin	Normal: ≥ 1st percentile MoM (n = 774,392)	5846 (6.0)	1.0 (Ref.)	1.0 (Ref.)
	Abnormal: < 1st percentile MoM (n = 10,235)	48 (5.4)	1.2 (0.9 to 1.6)	1.1 (0.8 to 1.5)
Low unconjugated estriol	Normal: ≥ 1st percentile MoM (n = 789,151)	5762 (5.9)	1.0 (Ref.)	1.0 (Ref.)
	Abnormal: < 1st percentile MoM (n = 10,212)	145 (8.8)	1.3 (1.1 to 1.5)	1.3 (1.1 to 1.5)
High dimeric inhibin-A	Normal: ≤ 99th percentile MoM (n = 90,761)	272 (4.0)	1.0 (Ref.)	1.0 (Ref.)
	Abnormal: > 99th percentile MoM (n = 1065)	9 (11.0)	2.7 (1.4 to 5.3)	2.4 (1.2 to 4.7)
Low pregnancy-associated plasma protein A	Normal: ≥ 1st percentile MoM (n = 387,651)	1045 (3.6)	1.0 (Ref.)	1.0 (Ref.)
	Abnormal: < 1st percentile MoM (n = 5748)	29 (6.8)	1.9 (1.3 to 2.7)	1.7 (1.2 to 2.5)

[a]Adjusted for maternal age (continuous), gravidity (1, 2+, missing), neighbourhood income quintile (1, 2, 3, 4, 5, missing), rural residence (rural, urban, missing), ethnicity (Asian, Black Caucasian, Hispanic, Oriental, other, missing), and gestational age (continuous) – each at the time of prenatal biochemical screening – as well as maternal diabetes mellitus, chronic hypertension, renal disease, tobacco/drug use and dyslipidemia within 365 days preceding the start of the index pregnancy, up to and including 365 days after the start of the index pregnancy (i.e. time zero). Censored on death or arrival at the end of study date of March 31, 2016.

Supplementary file 9. Description of the number of women who had at least 1 pregnancy with a measurement of a given serum analyte (column a), the percentage of those women who had 2 or more pregnancies in which that given analyte was measured (column b), and the percentage of those women with 2 or more abnormal results for that given analyte (column c).

	(a)	(b)	(c)
**Serum analyte**	**No. women with ≥ 1 pregnancy in which the serum analyte was measured**	**No. (%) of women in (a) with ≥ 2 pregnancies in which the analyte was measured**	**No. (%) of women in (b) who had ≥ 2 abnormal results for the given analyte**
Alphafetoprotein	1,125,431	267,708/1,125,431 (23.8)	2706/267,708 (1.0)
Human chorionic gonadotropin	1,092,685	259,570/1,092,685 (23.8)	3557/259,570 (1.4)
Unconjugated estriol	1,115,382	266,096/1,115,382 (23.9)	3755/266,096 (1.4)
Dimeric inhibin-A	98,708	6516/98,708 (6.6)	103/6516 (1.6)
Pregnancy-associated plasma protein A	503,720	98,263/503,720 (19.5)	1503/98,263 (1.5)

Supplementary file 10 (Additional analysis 5). Risk of the primary cardiovascular disease composite outcome of any hospitalization or revascularization for coronary artery, cerebrovascular or peripheral arterial disease, heart failure or dysrhythmia, arising ≥ 365 days after the start of the index pregnancy, in association with an abnormal cut-point of the 5th or 95th percentile of the multiple of the median (MoM) for a given serum analyte. This analysis also adjusts for the number of prior pregnancies with a given abnormal serum analyte, as described in column c of Supplementary file 9.

		Cardiovascular disease composite outcome		
**Abnormal serum analyte**	**Cut-points used to define normal and abnormal**	**No. (incidence rate per 10,000 person years)**	**Unadjusted hazard ratio (95% CI)**	**Adjusted hazard ratio (95% CI)[a]**
High alphafetoprotein	Normal: ≤ 95th percentile MoM (n = 763,716)	5600 (5.9)	1.0 (Ref.)	1.0 (Ref.)
	Abnormal: > 95th percentile MoM (n = 43,576)	420 (7.4)	1.2 (1.1 to 1.3)	1.2 (1.1 to 1.3)
Low total human chorionic gonadotropin	Normal: ≥ 5th percentile MoM (n = 741,491)	5670 (6.0)	1.0 (Ref.)	1.0 (Ref.)
	Abnormal: < 5th percentile MoM (n = 43,136)	224 (5.8)	1.3 (1.2 to 1.4)	1.2 (1.1 to 1.4)
Low unconjugated estriol	Normal: ≥ 5th percentile MoM (n = 756,958)	5332 (5.8)	1.0 (Ref.)	1.0 (Ref.)
	Abnormal: < 5th percentile MoM (n = 42,405)	585 (8.5)	1.3 (1.1 to 1.5)	1.3 (1.2 to 1.4)
High dimeric inhibin-A	Normal: ≤ 95th percentile MoM (n = 87,097)	251 (3.8)	1.0 (Ref.)	1.0 (Ref.)
	Abnormal: > 95th percentile MoM (n = 4729)	30 (8.3)	2.2 (1.5 to 3.2)	2.0 (1.4 to 2.9)
Low pregnancy-associated plasma protein A	Normal: ≥ 5th percentile MoM (n = 371,097)	990 (3.6)	1.0 (Ref.)	1.0 (Ref.)
	Abnormal: < 5th percentile MoM (n = 22,302)	84 (5.1)	1.4 (1.1 to 1.8)	1.3 (1.1 to 1.7)

[a]Adjusted for maternal age (continuous), gravidity (1, 2+, missing), neighbourhood income quintile (1, 2, 3, 4, 5, missing), rural residence (rural, urban, missing), ethnicity (Asian, Black Caucasian, Hispanic, Oriental, other, missing) and gestational age (continuous) – each at the time of the woman's last prenatal biochemical screening – as well as maternal diabetes mellitus, chronic hypertension, renal disease, tobacco/drug use and dyslipidemia within 365 days preceding the start of the woman's last pregnancy, up to and including 365 days after the start of the woman's last pregnancy. Also adjusted for the number of prior pregnancies with a given abnormal serum analyte.

Supplementary file 11 (Additional analysis 6). Comparison of 750,742 pregnancies with prenatal biochemical screening (screened cohort) and 750,742 pregnancies without biochemical screening (non-screened cohort), matched on year of delivery. One pregnancy was possible per woman. All data are presented as number (%) unless otherwise indicated.

**Characteristic**	**Screened cohort (N = 750,742)**	**Non-screened cohort (N = 750,742)**	**Standardized difference**
In the index pregnancy			
Mean maternal (SD) age, years	29.9 (5.3)	28.9 (5.9)	0.18
Income quintile (Q)			
Q1 (lowest)	170,988 (22.8)	184,721 (24.6)	-0.04
Q2	153,854 (20.5)	155,601 (20.7)	-0.01
Q3	150,552 (20.1)	148,776 (19.8)	0.01
Q4	149,474 (19.9)	142,983 (19.0)	0.02
Q5 (highest)	123,027 (16.4)	114,227 (15.2)	0.03
Unknown	2,847 (0.4)	4,434 (0.6)	-0.03
Residence			
Urban	694,690 (92.5)	644,616 (85.9)	0.22
Rural	55,078 (7.3)	105,757 (14.1)	-0.22
Unknown	974 (0.1)	369 (0.0)	0.03
Gravidity			
1	316,927 (42.2)	417,780 (55.6)	-0.27
≥ 2	423,400 (56.4)	332,962 (44.4)	0.24
Unknown	10,415 (1.4)	0 (0.0)	0.17
Pregnancy outcome			
Livebirth at ≥ 20 weeks' gestation	723,180 (96.3)	727,748 (96.9)	-0.03
Stillbirth at ≥ 20 weeks' gestation	3,684 (0.5)	4,476 (0.6)	-0.01
Miscarriage at < 20 weeks' gestation or ectopic pregnancy	3,146 (0.4)	22 (0.0)	0.09
Induced abortion at < 20 weeks' gestation	2,678 (0.4)	18,496 (2.5)	0.00
Unknown	18,054 (2.4)	0 (0.0)	0.22
Multifetal pregnancy	8,465 (1.1)	11,691 (1.6)	-0.04

Characteristic	Screened cohort (N = 750,742)	Non-screened cohort (N = 750,742)	Standardized difference
Year of screening/pregnancy outcome			
1993-2002	362,699 (48.3)	362,699 (48.3)	0.00
2003-2012	388,043 (51.7)	388,043 (51.7)	0.00
Conditions ≤ 365 days before, or up to 365 days after, the start of pregnancy			
Diabetes mellitus	41,185 (5.5)	42,796 (5.7)	-0.01
Chronic hypertension	24,896 (3.3)	43,584 (5.8)	-0.12
Dyslipidemia	8,216 (1.1)	11,259 (1.5)	-0.04
Renal disease	1,934 (0.3)	2,151 (0.3)	-0.01
Drug/alcohol/tobacco abuse	9,957 (1.3)	20,588 (2.7)	-0.10
Conditions at the time of a livebirth or stillbirth delivery			
Congenital or chromosomal anomaly	28,370 (3.9)	27,187 (3.7)	0.01
Preeclampsia/eclampsia	15,415 (2.1)	16,227 (2.2)	0.00
Gestational hypertension	22,832 (3.1)	23,173 (3.2)	0.00
Placental abruption	6,805 (0.9)	7,513 (1.0)	-0.01
Placental infarction	4,626 (0.6)	4,968 (0.7)	0.00
Conditions at the time of a livebirth delivery			
Preterm birth < 37 weeks' gestation	50,087 (6.9)	54,077 (7.4)	-0.02
Experienced the composite CVD outcome[a]			
No. events (incidence rate per 10,000 person-years)	6,504 (6.1)	5,901 (6.7)	--
Crude HR (95% CI)	1.0 (referent)	1.1 (1.1 to 1.1)	--
Adjusted HR (95% CI)[b]	1.0 (referent)	1.1 (1.0 to 1.1)	--

[a]Any hospitalization or revascularization for coronary artery, cerebrovascular or peripheral arterial disease, heart failure or dysrhythmia, arising ≥ 365 days after the start of the index pregnancy.

[b]Adjusted for maternal age (continuous), gravidity (1, 2+, missing), neighbourhood income quintile (1, 2, 3, 4, 5, missing), rural residence (rural, urban, missing) – each at the time of prenatal biochemical screening (screened cohort) or at the end of pregnancy (non-screened cohort) – as well as maternal diabetes mellitus, chronic hypertension, renal disease, tobacco/drug use and dyslipidemia within 365 days preceding the start of the index pregnancy, up to and including 365 days after the start of the index pregnancy (i.e. time zero). Censored on arrival at the end of study date of March 31, 2016.
